# Comparative Analysis of the Chemical Composition of Hemp and Linseed Varieties as Key Industrial Commodities

**DOI:** 10.3390/foods15122145

**Published:** 2026-06-14

**Authors:** Tomáš Taubner, Michaela Englmaierová, Marie Bjelková, Věra Skřivanová, Klára Bejčková, Tomáš Vít, Kateřina Růnová

**Affiliations:** 1 Department of Nutrition Physiology and Animal Product Quality, Institute of Animal Science, Přátelství 815, 104 00 Prague-Uhříněves, Czech Republic; englmaierova.michael@vuzv.cz (M.E.); skrivanova.vera@vuzv.cz (V.S.); bejckova.klara@vuzv.cz (K.B.); vit.tomas@vuzv.cz (T.V.); runova.katerina@vuzv.cz (K.R.); 2 Department of Legumes and Technical Crops, Agritec Plant Research Ltd., Zemědělská 2520/16, 787 01 Šumperk, Czech Republic; bjelkova@agritec.cz

**Keywords:** hemp seed, linseed, chemical composition, analysis, varieties

## Abstract

Hemp and linseed are nutritionally valuable commodities that exhibit considerable varietal differences in composition. Nutrient composition was evaluated in 12 hemp and 11 linseed varieties, including commercially cultivated varieties from the EU Common Catalogue and newly bred lines, to assess varietal variability. Field experiments were conducted under uniform agronomic conditions in the Czech Republic during a single growing season using field-block samples. Analyses included proximate composition (dry matter, crude protein, fat, fiber, ash), fatty acid and amino acid profiles, carotenoids, vitamins, and cannabinoid content. Statistical evaluation was performed using a General Linear Model with Duncan’s multiple range test (*p* < 0.05). Significant differences were observed across most parameters, indicating substantial inter-varietal variability under the experimental conditions. Fat content ranged from approximately 200 to 377 g/kg in both oilseeds, with lipids dominated by polyunsaturated fatty acids, particularly linoleic (n-6) and α-linolenic (n-3) acids. Hemp and linseed show potential as alternative plant protein sources in animal nutrition, but further digestibility and feeding studies are needed to confirm their suitability as partial soybean meal substitutes. Varietal selection may contribute to improved nutritional quality while influencing levels of undesirable constituents such as Δ^9^-THC in hemp.

## 1. Introduction

Hemp seed (*Cannabis sativa* L.) and linseed (*Linum usitatissimum* L.) are important agricultural commodities and valuable sources of nutrients with considerable potential in both human and animal nutrition. Hemp seeds typically contain approximately 30% lipids, 25% protein, and substantial amounts of dietary fiber, vitamins, and minerals [1]. Lipids represent the dominant fraction, with more than 80% polyunsaturated fatty acids (PUFAs) and a favorable n-6:n-3 ratio of approximately 3:1. In addition, hemp seeds contain γ-tocopherol, sterols (including β-sitosterol), and a wide range of bioactive compounds such as polyphenols, terpenoids, and trace amounts of cannabinoids, all of which contribute to antioxidant and anti-inflammatory properties [2,3]. Similarly, linseed is characterized by a high nutritional value, containing approximately 40% lipids, 20% protein, 10–20% dietary fiber, and around 4% ash [4]. It is one of the richest plant sources of n-3 fatty acids, particularly α-linolenic acid (ALA), which is associated with beneficial effects on cardiovascular and metabolic health [5]. Linseed also provides significant amounts of dietary fiber and lignans, which exhibit antioxidant activity and have been linked to protective effects against chronic diseases [6,7]. Due to their favorable nutritional profiles, both hemp seed and linseed have been widely incorporated into animal nutrition. Their inclusion in feed mixtures has been associated with improvements in production parameters, such as egg yield and quality, enhanced fatty acid profiles of animal products, reduced cholesterol levels, and improved nutrient [8,9,10,11]. Additional benefits include improved bone strength in laying hens [10,12].

Despite these well-documented nutritional benefits, the reported chemical composition of hemp and linseed is typically presented as average values, although it is strongly influenced by multiple factors, including environmental conditions, agronomic practices, and harvest timing. Among these, genotype (variety) is considered an important determinant of nutrient composition. However, most available studies focus on individual crops or are conducted under varying environmental conditions, which limits direct comparability and complicates the interpretation of varietal effects. Although studies such as that by Arango et al. [13] have evaluated a defined set of compositional parameters in hemp seed varieties, including proximate composition, basic fatty acid profile, amino acid profile, mineral content, and selected cannabinoids, a detailed, comprehensive and systematic comparison of both hemp and linseed varieties grown under similar environmental conditions is still lacking. Consequently, a research gap exists in the comparative evaluation of varietal differences in nutrient composition across these two nutritionally important oilseed crops under comparable growing conditions, which represents an important step toward identifying genotype-associated differences.

Therefore, the primary objective of this study was to evaluate inter-varietal differences in nutrient composition among commonly cultivated hemp and linseed varieties, most of which are included in the EU Common Catalogue of Varieties of Agricultural Plant Species and newly bred lines. The experiment was conducted under standardized field conditions at two locations in the Czech Republic, reducing but not eliminating the influence of environmental variability. A total of 12 hemp and 11 linseed varieties were analyzed in duplicates (independent biological replicates), allowing for a systematic comparison among varieties. The working hypothesis of this study was that (i) significant differences exist in nutrient composition among individual hemp and linseed varieties, and (ii) such differences represent varietal variability observed under the specific conditions of the present study and are statistically significant at *p* < 0.05. Furthermore, it was hypothesized that this variability may provide a basis for the targeted selection of varieties with improved nutritional profiles, pending validation across multiple growing seasons and environments. Hemp seed and linseed were selected for this study because both are recognized as valuable oilseed crops with similar applications in animal nutrition, where they are used as sources of protein, oil, and bioactive compounds. Previous studies have frequently evaluated these commodities within the same nutritional context, and they may be used either in combination or as partial alternatives to one another in feed formulations aimed at improving the fatty acid profile and nutritional quality of animal products. The analytical approach included comprehensive characterization of proximate composition (dry matter, crude protein, fat, dietary fiber, and ash), fatty acid profiles, amino acid composition, carotenoids, vitamins, and a broad spectrum of cannabinoids. This multi-level analysis enabled a detailed assessment of both nutritional and bioactive components across all evaluated varieties. The novelty of this study lies in the direct, side-by-side comparison of a relatively large set of commercially relevant hemp and linseed varieties grown under similar environmental conditions, enabling a more systematic assessment of varietal differences. In addition, this study provides an integrated dataset combining macronutrient composition with detailed biochemical profiling within a single experimental framework. Such a holistic and comparative approach to two major oilseed crops remains underrepresented in the current literature. From an applied perspective, the results of this study provide compositional insights that may support varietal selection in animal nutrition and suggest a potential for further evaluation of these crops as alternative plant protein sources, including their possible use in partial replacement of conventional protein sources such as soybean meal, pending confirmation by digestibility and feeding studies. Moreover, the findings allow the preliminary identification of varieties exhibiting more favorable compositional profiles under the studied conditions, including higher levels of desirable nutrients (e.g., PUFAs such as linoleic acid and ALA, essential amino acids such as lysine, methionine, and leucine, and antioxidant compounds such as tocopherols and carotenoids) and lower levels of undesirable compounds (e.g., Δ^8^-THC, Δ^9^-THC, or Δ^9^-THCA). However, further multi-year and multi-location evaluations are required to confirm the stability of these traits and to better distinguish the contributions of genotype and environmental factors.

## 2. Materials and Methods

### 2.1. Analysis of Hemp Seed and Linseed Varieties

Twelve hemp seed varieties and eleven linseed varieties (Table 1) were selected for comparative analysis. All hemp seed and linseed varieties, with the exception of the Spring, Winter, and Bethune varieties, are registered in the EU and listed in the Common Catalog of Varieties of Agricultural Plant Species. The Spring (genotype AGT 1/2020) and Winter (genotype AGT 2/2020) varieties were bred by Agritec Plant Research Ltd. (Šumperk, Czech Republic).

**Figure 1:**
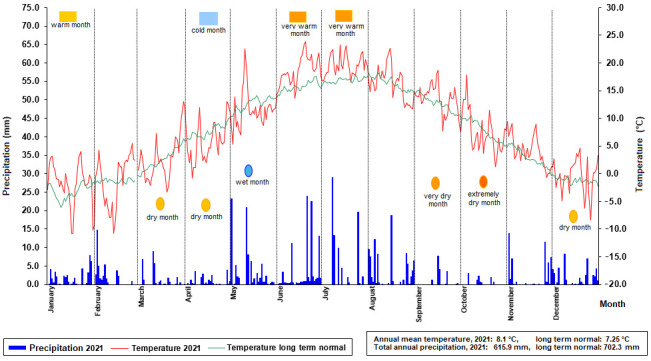
Average daily air temperature and daily precipitation totals, Šumperk site in 2021, long-term normal.

All analyzed varieties were cultivated and harvested in 2021 under recorded climatic conditions (Table 2, Figure 1) by Agritec Plant Research s.r.o. at the Šumperk site (49°58′34.1″ N, 16°57′55.3″ E) and Vikýřovice ’U letiště‘ site (49°57′59.10″ N, 17°00′48.28″ E). All varieties were grown at both locations, allowing the assessment of varietal differences, location effects, and their interaction under the specific environmental conditions of the 2021 growing season. The Šumperk study area is characterized by a moderately warm climate, with an average annual precipitation of 692 mm and a mean annual temperature of 7.25 °C. Precipitation occurs most frequently during the months of June, July, and August. The site is dominated by pseudogley soils, occurring mainly on gentle slopes with omnidirectional exposure and a total skeletal content of up to 10%. The soils are deep, located in a moderately warm and moderately humid climatic region, and are characterized by low productivity. The prevailing soil type is gleyic chernozem (HNg).

The Šumperk experimental site is characterized by loamy soil with a gleyed soil-forming substrate. The soil is well workable and moderately supplied with nutrients, with a pH of 6.5 and a medium humus content. The average topsoil depth is approximately 28 cm. The K:Mg ratio is favorable, and no magnesium nutrition deficiencies are expected. The Vikýřovice experimental site, known as ‘U letiště’ (49°57′59.10″ N, 17°00′48.28″ E), is classified under the evaluated soil-ecological unit 7.43.00. According to Decree No. 48/2011 Coll. on the determination of agricultural land protection classes, this site falls within Protection Class II of the Agricultural Land Fund. The climatic region is classified as Region 7-moderately warm and humid (MT4). The dominant soil types are gleyic luvic chernozem (HNlg) and gleyic luvisol (LUg). The results of the agrochemical soil analysis are presented in Table 3.

All hemp and linseed varieties were cultivated at both experimental locations. The field experiment was established using a uniform plot layout for all varieties at both locations. Each variety was grown in plots consisting of three rows, each 10 m in length, with a row spacing of 0.50 m. Standard agronomic practices, including soil preparation, fertilization, sowing, weed control, and crop management, were applied according to the methodology routinely used by Agritec Plant Research s.r.o. For biological replication, two independent biological samples were obtained for each variety at each location. Each biological replicate consisted of a composite (pooled) seed sample prepared independently from plant material harvested from separate sections of the experimental plot. These samples were processed independently throughout the analytical procedure and were treated as biological replicates for statistical evaluation. Harvesting was performed at full seed maturity, at crop-specific maturity stages defined for each variety (Table 1). Following harvest, seeds from each biological replicate were cleaned, homogenized, and prepared for laboratory analyses. Each biological replicate was analyzed independently, and all analytical determinations were performed in duplicate (technical replicates). Mean values from technical replicates were used for statistical analysis. The experimental design included two locations and two biological replicates per variety. Data from both locations were included in the statistical analyses, and varietal differences were evaluated across locations.

### 2.2. Sample Preparation

All samples were stored at low temperature (4 °C) in a refrigerator. Whole hemp or linseeds (including the hull) from individual varieties were ground prior to analysis using laboratory grinders specific to each method, namely the Retsch GM200 and Retsch MM400 (Retsch GmbH, Haan-Gruiten, Germany).

### 2.3. Chemical Composition Analysis

Dry matter, crude protein, fat, dietary fiber, and ash (g/kg) were analyzed according to international Association of Official Analytical Chemists (AOAC) standard procedures [14]. Dry matter, crude protein, crude fat, crude fiber, and ash contents were determined according to AOAC Official Methods 934.01, 984.13, 920.39, 978.10, and 942.05, respectively. Data were expressed as g/kg fresh weight. The dry matter of the diets was determined by drying to constant weight at 105 °C in an oven (Memmert ULM 500; Memmert, Schwa-bach, Germany). The crude protein content was measured using a Kjeltec Auto 1030 in-strument (Tecator, Höganäs, Sweden). The fat content was determined upon extraction with petroleum ether using a 1045 Soxtec Tecator extraction system (Tecator). Crude fiber content was measured using a Fibertec 2010 fiber analyzer (Tecator, Höganäs, Sweden). The nitrogen-free extract (NFE, g/kg) was calculated using the following Formula (1):

(1) NFE (g/kg) = (100 − (moisture + crude protein + fat + dietary fiber + ash)) × 10

### 2.4. Fatty Acid Composition

The fatty acid composition of the hemp seeds and linseed was determined following the extraction of total lipids using chloroform–methanol [15]. The alkaline trans-methylation of the fatty acids was performed as described by Raes et al. [16]. Gas chromatographic analysis of the fatty acid methyl esters (FAMEs) was performed using an HP 6890 chromatograph (Agilent Technologies, Inc., Santa Clara, CA, USA) equipped with a 60 m DB-23 capillary column (150–230 °C) and a flame ionization detector. Split injections were performed using an Agilent autosampler, with 1 μL FAME samples in hexane injected at a 1:40 split ratio. Separation was achieved using the following column temperature program: initially, the column was held at 60 °C for 7 min, then ramped at 20 °C/min to 110 °C and held for 4 min, followed by a ramp at 10 °C/min to 120 °C and held for 4 min; then 15 °C/min to 170 °C; 2 °C/min to 210 °C and held for 13.5 min; and finally 40 °C/min to 230 °C and held for 7 min. Fatty acids were identified by comparing their retention times with those of reference standards PUFA No.1 (cat. no. 47033), PUFA No.2 (cat. no. 47015-U), PUFA No.3 (cat. no. 47085-U), and a 37-component FAME mixture (Supelco, Bellefonte, PA, USA). For quantification, nonadecanoic acid (C19:0; cat. no. N5252, Sigma-Aldrich, Prague, Czech Republic) was used as an internal standard. The fatty acid composition was expressed as mg/kg or g/kg fresh weight.

### 2.5. Amino Acid Composition

Amino acids were determined according to AOAC [14] Official Method 982.30 with minor modifications. The amino acids were determined by hydrolyzing the proteins in 6 M HCl at 110 °C for 23 h. Sulfur-containing amino acids were oxidized with peroxyformic acid for 16 h prior to hydrolysis. After evaporation, the hydrolysate was dissolved in a citrate buffer (pH 2.2) and diluted to an appropriate concentration. Amino acids were analyzed using an Ingos AAA 500 device (Ingos s.r.o., Prague, Czech Republic). The analysis involved separation on an ion-exchange column with citrate buffers, postcolumn derivatization with ninhydrin reagent, and spectrophotometric detection at 440 and 570 nm. Identification and quantification of amino acids were performed using a certified amino acid standard mixture as an external standard. Quantification was based on individual calibration curves established for each amino acid from standard solutions of known concentrations. Chromatograms were evaluated by comparison of retention times and peak areas with those of the external standards. The amino acid composition was expressed as g/kg fresh weight. Tryptophan was not included in the amino acid analysis due to its well-known instability under conventional acid hydrolysis conditions, during which it is readily degraded and therefore cannot be reliably quantified using the applied analytical procedure.

### 2.6. Analysis of Carotenoids and Vitamins

The lutein and zeaxanthin contents were analyzed using a high-performance liquid chromatography (HPLC) system (VP series; Shimadzu, Kyoto, Japan) according to a modified version of the method proposed by Cui et al. [17]. The HPLC system was equipped with a diode array detector (DAD) and a YMC C30 column (250 × 4.6 mm, 5 µm; YMC Co., Ltd., Kyoto, Japan). The composition of the mobile phase was dichloromethane:acetonitrile:methanol (20:30:50, *v*/*v*/*v*), the flow rate 1 mL/min and column temperature 30 °C. The sample was saponified with 60% aqueous KOH and extracted with n-hexane:ethyl ether:cyclohexane (40:40:20, *v*/*v*/*v*). The detection wavelength was 446 nm. The concentrations of α-tocopherol, γ-tocopherol, δ-tocopherol, and β-carotene (mg/kg) in the analyzed seeds were determined using the same HPLC system (VP series; Shimadzu, Kyoto, Japan) equipped with a diode array detector (HPLC-DAD) and Phenomenex Synergi 4 μm Fusion-RP 80 Å column (150 × 4.6 mm, 4 μm; P/No. 00F-4424-E0, Torrance, CA, USA) using methanol as the mobile phase after alkaline saponification with 60% aqueous KOH and extraction with diethyl ether, according to the European standards (European Committee for Standardisation: EN 12822-1, EN 12823-1, and EN 12823-2) [18,19,20]. The method employed a gradient flow program, where the solvent composition remained constant and the flow rate was adjusted from 0.6 mL/min to 1.5 mL/min and back to 0.6 mL/min. A 50 μL sample injection volume was used. Quantification was performed using individual calibration curves for each compound. Detection wavelengths (λ) were set as 325 nm for retinol, 292 nm for α-tocopherol, 296 nm for γ-tocopherol, 296 nm for δ-tocopherol, and 450 nm for β-carotene. The concentrations of carotenoids and vitamins were expressed as mg/kg fresh weight.

### 2.7. Analysis of Cannabinoids

All analytical procedures were carried out exactly as described in previous authors’ published methods [21,22], which provide complete chromatographic and instrumental conditions. Cannabinoids were analyzed using the method proposed by Taubner and Czauderna [22] for the quantification of 16 neutral and acidic forms of cannabinoids in various biological materials by reversed-phase C18 HPLC (RP-C18-HPLC). The compounds included Δ^9^-tetrahydrocannabinol (Δ^9^-THC), Δ^9^-tetrahydrocannabinolic acid (Δ^9^-THCA), Δ^8^-tetrahydrocannabinol (Δ^8^-THC), cannabidiol (CBD), cannabidiolic acid (CBDA), cannabinol (CBN), cannabinolic acid (CBNA), cannabigerol (CBG), cannabigerolic acid (CBGA), cannabichromene (CBC), cannabichromenic acid (CBCA), Δ^9^-tetrahydrocannabivarin (THCV), Δ^9^-tetrahydrocannabivarinic acid (THCVA), cannabidivarin (CBDV), cannabidivarinic acid (CBDVA), and cannabicyclol (CBL). All ground samples were extracted using methanol and n-hexane in four sequential extraction steps. The extracts were injected into the HPLC system on C18 columns with gradient elution and a diode array detector (DAD). To identify and verify analyzed cannabinoids in linseed, the method of Czauderna et al. [21], which is based on the GC–MS determination of cannabinoids, was used. The concentrations of cannabinoids in the hemp seed and linseed samples were expressed as mg/kg fresh weight.

### 2.8. Statistical Analysis

The data were analyzed in duplicate using analysis of variance (ANOVA) within the general linear model (GLM) framework in SAS software version 9.3 (SAS Institute Inc., Cary, NC, USA) [23]. The statistical model included the fixed effects of variety and location, as well as their interaction (variety × location), according to the following model (2):

(2) y = μ + V + L + (V × L) + ε

where y is the observed trait, μ is the overall mean, V represents the effect of variety, L represents the effect of location, V × L represents the interaction between variety and location, and ε is the random error term.

When the interaction effect was not statistically significant, it was removed from the final model and only the main effects were interpreted. Differences among varieties were evaluated using Duncan’s multiple range test applied to model-adjusted means. This post hoc procedure was selected as it is widely used in agricultural and plant breeding studies for genotype comparison under field experimental conditions, where the objective is to detect meaningful biological differences among a relatively large number of varieties. Duncan’s test provides higher sensitivity in detecting differences among means compared to more conservative procedures and is therefore frequently applied in varietal screening experiments. Although Duncan’s test is less conservative with respect to control of type I error in multiple comparisons, its use is considered appropriate in exploratory agronomic studies focused on identifying varietal differences under controlled experimental conditions. The results are presented as mean ± standard error of the mean (SEM). Statistical significance was declared at *p* < 0.05.

## 3. Results and Discussion

### 3.1. Proximate Composition of Hemp Seed and Linseed

The basic chemical compositions of different varieties of hemp seed (Table 4) and linseed (Table 5) were evaluated.

The dry matter content of both matrices exceeded 900 g/kg, with an average of approximately 950 g/kg, indicating a low moisture content and consequently good storage stability of both oilseeds.

The crude protein content averages 241 g/kg in hemp seed and 199 g/kg in linseed, indicating that hemp represents a richer protein source under the studied conditions. From a nutritional perspective, this difference may be relevant for plant-based diet formulation, where protein density is a limiting factor. The higher protein level in hemp is consistent with its role as a source of highly digestible storage proteins.

Substantial differences were observed in fat content between hemp and linseed, as well as among individual varieties. In hemp seed, fat content ranged from 200 g/kg in the Białobrzeskie variety to 298 g/kg in Dioica 88, with a mean value of 255 g/kg. In contrast, linseed exhibited significantly higher fat content, averaging 342 g/kg, ranging from 289 g/kg (Astella) to 377 g/kg (Koral). These differences are consistent with previously reported variability in oil content and fatty acid composition across genotypes [24]. From a technological and economic perspective, this higher lipid content may contribute to increased oil yield, making linseed more suitable for oil production and lipid-based nutritional applications. Conversely, the use of hemp seed for such applications requires targeted selection of high-oil cultivars.

Total dietary fiber content was markedly higher in hemp seed (237 g/kg) than in linseed (154 g/kg), representing an average difference of approximately 100 g/kg. This elevated fiber level in hemp may have nutritional relevance, particularly in terms of gastrointestinal health, glycemic response modulation, and satiety. Among linseed varieties, fiber ranged from 92 g/kg (Raciol) to 190 g/kg (Bukoz); however, even the highest value remained lower than that of the lowest hemp variety (Futura 75), highlighting a clear compositional difference under the studied conditions.

Total mineral contents were comparable (47.1 g/kg in hemp and 37.0 g/kg in linseed), differing by approximately one percentage point, suggesting similar contributions to micronutrient intake.

NFE accounted for approximately 200 g/kg in both matrices. Overall, the compositional profiles (Table 4 and Table 5) fall within ranges reported in the literature [1,4], with only minor deviations. Importantly, the observed inter-varietal variability provides preliminary evidence supporting further evaluation of breeding and selection strategies under multi-year and multi-location conditions to confirm the stability of the observed nutritional traits.

### 3.2. Fatty Acid Composition and Nutritional Implications

The fatty acid profiles of hemp seed and linseed were comprehensively analyzed, including saturated fatty acids (SFAs; Table 6 and Table 7), unsaturated fatty acids (Table 8 and Table 9), and total fatty acid group distributions (Table 10 and Table 11).

PUFAs predominated in both seed types, indicating their high nutritional value under the studied conditions. Linoleic acid (C18:2, n-6) was the major fatty acid in both matrices, with an average content of 171.8 g/kg in hemp seed and approximately 90.7 g/kg in linseed. While hemp exhibited a relatively narrow variability range (133.5–225.7 g/kg), linseed showed substantially broader variation (57.5–233.1 g/kg), suggesting stronger varietal influence on fatty acid composition. ALA (C18:3, n-3), a physiologically essential n-3 fatty acid, was present at moderate levels in hemp (average 50.2 g/kg) but was markedly higher in linseed (average 251.9 g/kg). This difference is nutritionally critical, as ALA serves as a precursor for long-chain n-3 fatty acids involved in anti-inflammatory pathways and cardiovascular protection [25,26]. Extreme variability was observed among linseed varieties, ranging from 6.7 g/kg in Agriol to 344.0 g/kg in Bukoz, which may reflect differences in breeding history and selection. *Agriol* represents a low-ALA genotype, whereas Agram and Raciol are medium-level varieties, suggesting that fatty acid composition is a strongly varietal-influenced trait under studied conditions.

The balance between n-6 and n-3 fatty acids is a key determinant of nutritional quality. The average n-6/n-3 ratio was 3.56:1 in hemp and 1.18:1 in linseed. The latter is considered more favorable from a human health perspective, as lower ratios are associated with reduced risk of chronic inflammatory diseases. However, the Agriol variety exhibited an unfavorable ratio of 8.97:1, combined with low PUFA and n-3 content, illustrating that not all linseed varieties are nutritionally equivalent.

Among other major fatty acids, oleic acid (C18:1) and palmitic acid (C16:0) were present in comparable amounts across both matrices [27,28]. Palmitic acid was the dominant saturated fatty acid (SFA) (20.6 g/kg in hemp; 28.7 g/kg in linseed), followed by stearic acid (C18:0) (6.0 g/kg and 7.8 g/kg, respectively). SFAs accounted for approximately 10% of total fatty acids, which is nutritionally favorable, as lower SFA intake is associated with improved cardiovascular outcomes. The presence of γ-linolenic acid (C18:3, n-6), a bioactive metabolite, further enhances the functional value of hemp and linseed oils [29], as it participates in anti-inflammatory and immunomodulatory pathways. Statistically significant differences among varieties (*p* < 0.05; Table 6, Table 7, Table 8, Table 9, Table 10 and Table 11) are therefore not only analytically relevant but also biologically relevant under the studied conditions, as they directly influence lipid metabolism, oxidative stability, and health-related functionality. These findings support the potential for targeted selection of cultivars based on fatty acid composition, enabling optimization for both nutritional quality and industrial oil production [30].

The differences between lipid determination using AOAC gravimetric methods and fatty acid analysis by GC-FAME arise primarily from the distinct analytical principles and the different definitions of what is being measured. AOAC methods, such as Soxhlet extraction, determine “crude fat,” which represents the mass of lipids that can be extracted from the sample matrix using non-polar organic solvents. This approach is highly dependent on extraction efficiency, solvent selection, and the structural properties of the sample matrix. In plant seeds such as hemp or flax, incomplete extraction may occur, particularly for polar lipid fractions (e.g., phospholipids and glycolipids) or lipids tightly bound within cellular structures. As a result, the AOAC method may underestimate the total lipid content. In contrast, GC analysis of fatty acid methyl esters (FAME), performed after prior transesterification of lipids, provides a detailed profile of individual fatty acids released from all lipid classes, including triglycerides and a portion of polar lipids that may not be fully recovered by solvent extraction. Therefore, GC-FAME results represent the total potential fatty acid content rather than gravimetrically defined extractable fat. Another key difference is that AOAC methods quantify the entire lipid molecule, including the glycerol backbone in triglycerides, whereas GC-FAME quantifies only the fatty acid fraction after cleavage of the glycerol moiety. Consequently, the two approaches are fundamentally based on different analytical targets and are not directly comparable in a strict quantitative sense. Additional discrepancies may arise from methodological factors such as incomplete extraction, oxidative degradation of unsaturated fatty acids during sample preparation, variability in transesterification efficiency, and the use of conversion factors or internal standards. Due to these considerations, GC-FAME analysis may yield higher values than AOAC crude fat determination. This discrepancy does not necessarily indicate an analytical error, but rather reflects the broader coverage of lipid-bound fatty acids captured by GC methods and the inherent limitations of gravimetric extraction techniques.

### 3.3. Protein Quality and Amino Acid Composition

Hemp seeds are recognized as a source of high-quality protein with excellent digestibility [31,32]. Proteomic analyses have identified 181 proteins, predominantly albumins (250–370 g/kg) and edestin (67–75 g/kg) [33]. Edestin is particularly rich in arginine and glutamic acid and provides a balanced profile of essential amino acids [32,34], which is critical for human nutrition. Linseed proteins, composed mainly of albumins and globulins, also represent a valuable plant protein source [35,36]. However, protein quality is influenced by multiple factors, including genotype, environmental conditions, and processing [37], which can alter both amino acid composition and digestibility.

The measured amino acid profiles (Table 12 and Table 13) demonstrated a broad and balanced composition in both hemp and linseed. Significant varietal differences were observed for most amino acids (*p* < 0.05), indicating varietal-associated differences in seed protein composition. Nevertheless, the overall amino acid patterns remained broadly similar under the studied conditions. In both hemp and linseed, glutamic acid + glutamine represented the predominant amino acid fraction, ranging from 34.4–44.1 g/kg seed in hemp and 33.8–39.9 g/kg seed in linseed. Aspartic acid + asparagine and arginine were also among the most abundant amino acids. Arginine concentrations ranged from 20.7 to 26.3 g/kg seed in hemp and from 16.6 to 19.7 g/kg seed in linseed, whereas leucine varied between 12.9 and 16.5 g/kg seed in hemp and 10.3 and 12.0 g/kg seed in linseed.

Although both crops exhibited a balanced amino acid composition and contained all essential amino acids, several species-specific differences were evident. Hemp seeds generally contained higher concentrations of arginine and branched-chain amino acids, particularly leucine, whereas linseed was characterized by higher concentrations of glutamic acid + glutamine and proline. These differences are in line with the reported storage protein composition of both species and may influence their potential nutritional and functional properties. Nevertheless, the amino acid profiles of both hemp and linseed indicate high-quality plant proteins with the potential to complement each other in food and feed applications. In addition, the higher total protein concentration observed in hemp seeds may further enhance their nutritional value, while linseed provides a similarly balanced amino acid spectrum despite its lower protein content.

The amino acid composition expressed on a protein basis (g/kg protein) enabled comparison of the intrinsic amino acid profiles among varieties and species independently of differences in total protein concentration (Supplementary Tables S1 and S2). The results indicated relatively similar amino acid patterns within each crop species, suggesting that amino acid composition is strongly associated with species-specific storage protein composition. Nevertheless, several consistent differences between hemp and linseed proteins were observed. Hemp varieties contained higher concentrations of several nutritionally important amino acids, particularly arginine, leucine, isoleucine, valine, and phenylalanine, whereas linseed proteins were characterized by higher proportions of glutamic acid + glutamine, proline, glycine, and serine. The elevated arginine content of hemp protein is consistent with previous reports identifying hemp seed as a particularly rich source of this amino acid among plant proteins [32].

Among hemp varieties, glutamic acid + glutamine represented the predominant amino acid fraction, ranging from 141.6 to 178.5 g/kg protein, followed by arginine (91.8–116.6 g/kg protein) and aspartic acid + asparagine (87.7–106.5 g/kg protein). Lysine concentrations ranged from 28.7 to 35.6 g/kg protein, leucine from 54.7 to 66.8 g/kg protein, and methionine from 20.8 to 24.9 g/kg protein. While differences among cultivars were generally modest after normalization to protein content, varieties such as Carmagnola and Białobrzeskie tended to exhibit higher concentrations of several amino acids, whereas Santhica 27, Santhica 70, and Fibror 79 frequently showed lower values. These results are in agreement with the characteristic amino acid profile of hemp seed proteins, which are dominated by edestin and albumin storage proteins and are known for their high arginine content [32,34]. In linseed, glutamic acid + glutamine was also the dominant amino acid group, occurring at even higher concentrations than in hemp (174.0–201.0 g/kg protein). Arginine content ranged from 77.9 to 96.4 g/kg protein, while lysine varied between 31.9 and 47.7 g/kg protein. The varieties Floral and Szafir generally exhibited higher concentrations of several amino acids, whereas Koral tended to show lower values for most amino acids.

From a nutritional perspective, one of the most notable distinctions was the higher arginine concentration in hemp protein, with a mean value of 104.1 g/kg protein compared with 87.2 g/kg protein in linseed. Arginine plays important roles in nitric oxide synthesis, immune function, and protein metabolism, and the higher concentration observed in hemp protein may contribute to its nutritional value. Hemp proteins also contained higher concentrations of branched-chain amino acids (valine, isoleucine, and leucine), which are involved in muscle protein synthesis and metabolic regulation. Similar observations were reported by House et al. [32], who described hemp protein as a plant protein source with a favorable amino acid composition. Despite these differences, both crops exhibited a balanced amino acid profile and contained all essential amino acids. The relatively low concentrations of sulfur-containing amino acids, particularly methionine (20.8–25.8 g/kg protein in hemp and 20.3–22.9 g/kg protein in linseed), suggest that these amino acids may represent the primary limiting components of protein quality. Nevertheless, the overall amino acid composition confirms that both hemp and linseed proteins represent valuable plant protein sources. The observed varietal differences further indicate that cultivar selection may provide opportunities for optimizing specific nutritional characteristics, particularly in relation to arginine-rich hemp varieties and linseed genotypes with enhanced lysine content. Overall, the present results support previous findings that hemp and flaxseed possess distinct but complementary amino acid profiles, which may be exploited for targeted food and feed applications [32].

### 3.4. Tocopherols and Carotenoids as Lipid-Protective Bioactive Compounds

Hemp and linseeds are also valued for their bioactive compound content, including vitamin E (tocopherols), with a tocopherol profile characterized by the predominance of γ-tocopherol. According to Wu et al. [38], the average γ-tocopherol content in linseed is approximately 410 mg/kg dry matter, accounting for more than 50% of the total tocopherol content, whereas α-tocopherol is present only at concentrations of several tens of mg/kg. In addition to their physiological roles, tocopherols have been associated with a reduced risk of cardiovascular disease, cancer, and age-related macular degeneration [39]. The concentration and composition of tocopherols are influenced by cultivar [24]. A similar distribution of individual tocopherols has been reported for hemp seeds. Jiang et al. [40] reported γ-tocopherol as the dominant form in hemp seeds (216.8 mg/kg), followed by α-tocopherol (18.2 mg/kg), δ-tocopherol (12.0 mg/kg), and β-tocopherol (1.6 mg/kg). γ-Tocopherol is readily absorbed and accumulates in human tissues, where it exhibits anti-inflammatory properties [40]; however, its antioxidant activity is generally considered lower than that of α-tocopherol [41]. Owing to the high content of unsaturated fatty acids in hemp and linseeds, tocopherols play a critical role as antioxidants, protecting these lipids from oxidative degradation.

Our measured data (Table 14 and Table 15) indicate a higher overall tocopherol content and consequently higher levels of all the quantified tocopherol forms in hemp seeds than in linseeds. The average concentrations in hemp seeds were 13.8 mg/kg for α-tocopherol, 170.0 mg/kg for γ-tocopherol, and 24.7 mg/kg for δ-tocopherol, whereas in linseeds, the corresponding values were 1.36 mg/kg, 126.0 mg/kg, and 6.41 mg/kg, respectively. In the present dataset, the δ-tocopherol concentrations exceeded those of α-tocopherol. Significant differences in varieties were also observed, with the highest total tocopherol content detected in Dioica 88 (a hemp variety) and in Floral (a flax variety), followed by Agriol. In addition to tocopherols, hemp and linseeds contain bioactive carotenoids, including β-carotene, lutein, and zeaxanthin, which play important roles in human health because of their antioxidant and physiological functions [42,43,44].

β-Carotene serves as a provitamin A compound that can be enzymatically converted to retinol, which is essential for vision, immune function, and cellular health. It contributes to visual acuity, helps prevent night blindness, and has been associated with reduced oxidative modification of LDL cholesterol, suggesting a potential role in cardiovascular health and chronic disease risk reduction [44]. Lutein and zeaxanthin are xanthophyll carotenoids that selectively accumulate in the macula of the retina, where they absorb high-energy blue light and protect retinal tissues from photochemical damage and oxidative stress. Their intake has been associated with a reduced risk of age-related macular degeneration and other ocular disorders, and they may also exert anti-inflammatory and neuroprotective effects through their ability to scavenge reactive oxygen species [42,43]. The analytical data confirm the presence of these carotenoids in both hemp and linseeds. In hemp seeds, β-carotene is the predominant carotenoid, accompanied by lutein and zeaxanthin, collectively contributing to the antioxidant capacity of the seed matrix [45]. Linseeds also contain β-carotene, whose content markedly varies among varieties, suggesting a potential role in protecting seed lipids from oxidative deterioration [46]. The present results again indicate a higher concentration of these bioactive compounds in hemp seeds than in linseeds. Across all the hemp varieties, the average concentrations were 0.304 mg/kg for β-carotene, 30.9 mg/kg for lutein, and 1.00 mg/kg for zeaxanthin. In linseeds, the corresponding values were 0.128 mg/kg, 2.82 mg/kg, and 0.572 mg/kg, respectively, with particularly pronounced differences observed for lutein. The presence of these antioxidants is also technologically important, as they enhance lipid stability and extend shelf life by reducing oxidative deterioration. Varietal differences (e.g., Earlina 8 FC, Futura 75, Bethune) suggest the potential for selecting cultivars with enhanced functional properties.

### 3.5. Cannabinoid Content and Regulatory Relevance

Various varieties of hemp seed (Table 16) and linseed (Table 17) were analyzed using the RP-C18-HPLC-DAD method described by Taubner et al. [22], enabling the identification of 16 distinct cannabinoids. In addition, the total cannabinoid content was calculated as the sum of 16 major and most frequently occurring cannabinoids. Hemp seeds contain only trace amounts of cannabinoids [47], as cannabinoids are concentrated primarily in the flowers and leaves rather than in the seeds. The analysis confirmed that hemp seeds contain very low levels of cannabinoids, including nonpsychotropic CBD and Δ^9^-THC [48,49]. Industrial hemp varieties regulated for food use ensure minimal THC content, rendering hemp seeds safe for consumption without psychoactive effects. Although hemp seeds are a valuable source of nutrients such as proteins, lipids, and vitamins, they are not a primary source of cannabinoids for medicinal purposes [50]. Within the European Union, the content of Δ^9^-THC in hemp seeds and foods derived from them is regulated by the legally binding maximum limits established by Commission Regulation (EU) 2023/915 [51], which amends and consolidates Commission Regulation (EC) No. 1881/2006 on maximum levels for certain contaminants in foodstuffs. For hemp seeds (*Cannabis sativa* L.), including both whole and mechanically processed forms (e.g., crushed or ground seeds), the maximum permitted level is 3.0 mg/kg Δ^9^-THC equivalents. This limit applies to the sum of Δ^9^-THC and its acidic precursor, Δ^9^-THCA, with the Δ^9^-THCA content converted to Δ^9^-THC equivalents using a conversion factor of 0.877 to account for differences in molecular weight. For hemp seed oil, the maximum allowable concentration is 7.5 mg/kg Δ^9^-THC equivalents, reflecting the lipophilic nature of cannabinoids and their potential concentration during oil pressing and processing. These limits apply exclusively to food products and are independent of agricultural THC thresholds for hemp cultivation regulated under the EU Common Agricultural Policy. Food-related limits aim to protect consumers from potential psychoactive effects while recognizing that hemp seeds are not primary sites of cannabinoid biosynthesis and that their cannabinoid content is largely due to surface contamination by resin originating from other plant tissues [47,49,52].

The analyzed hemp seed varieties (Table 16) exhibited considerable variation in CBD content. The total CBD concentration ranged from 3.9 to 82.1 mg/kg, with an average of 29.6 mg/kg. The lowest CBD content was observed in the *USO 31* variety. Δ^9^-THC was detected in only four varieties, with the highest concentration recorded in Finola. CBD concentrations were the highest in Felina 32 (27.48 mg/kg), Finola (17.24 mg/kg), and Futura *75* (10.98 mg/kg). Similarly, CBDA concentrations were also highest in these varieties, albeit in a different order: Finola (54.8 mg/kg), Futura 75 (53.6 mg/kg), and Felina (29.5 mg/kg). Of particular interest were the contents of CBG and CBGA, which are typically less than 1 mg/kg in most varieties. However, the Santhica 27 and Santhica 70 varieties presented markedly higher concentrations of CBG (2.936 and 2.453 mg/kg, respectively) and CBGA (10.65 and 11.81 mg/kg, respectively). Santhica varieties are specifically bred for a higher CBG content and an almost negligible THC content. These differences largely reflect the cannabinoid composition of the entire plant, as the cannabinoid content detected in seeds corresponds to that of the whole plant. Consequently, individual varieties differ substantially. Analyses of whole-plant biomass of selected varieties such as Białobrzeskie, Felina 32, Futura 75, and Santhica 27 by Paulová et al. [53] demonstrated that the levels of major cannabinoids (CBD, CBDA, CBG, and CBGA) closely corresponded to those measured in the seeds. This observation is consistent with studies indicating that cannabinoids detected in seeds primarily originate from external contamination from other plant parts rather than endogenous biosynthesis within the seed itself [52].

**Figure 2:**
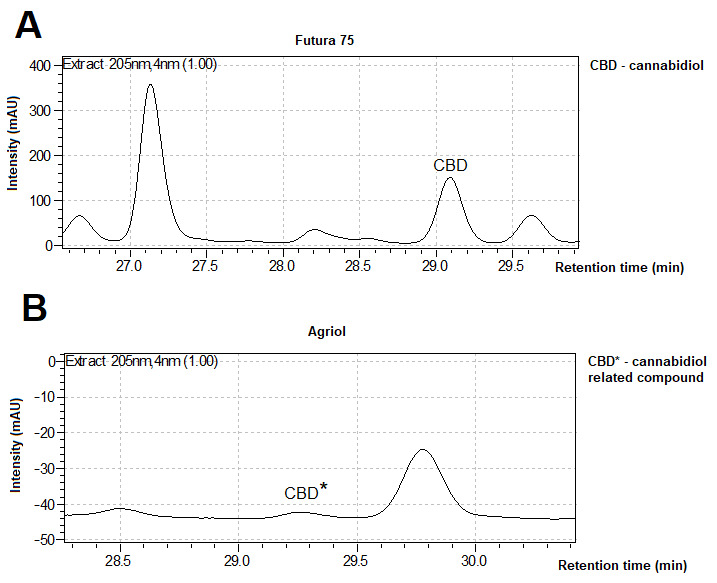
Comparison of high-performance liquid chromatography chromatograms (205 nm) of the hemp seed variety Futura 75 (**A**) and the linseed variety Agriol (**B**), showing the visible cannabidiol peak in the hemp seed variety and a cannabidiol-like compound (CBD*) in the linseed variety.

**Figure 3:**
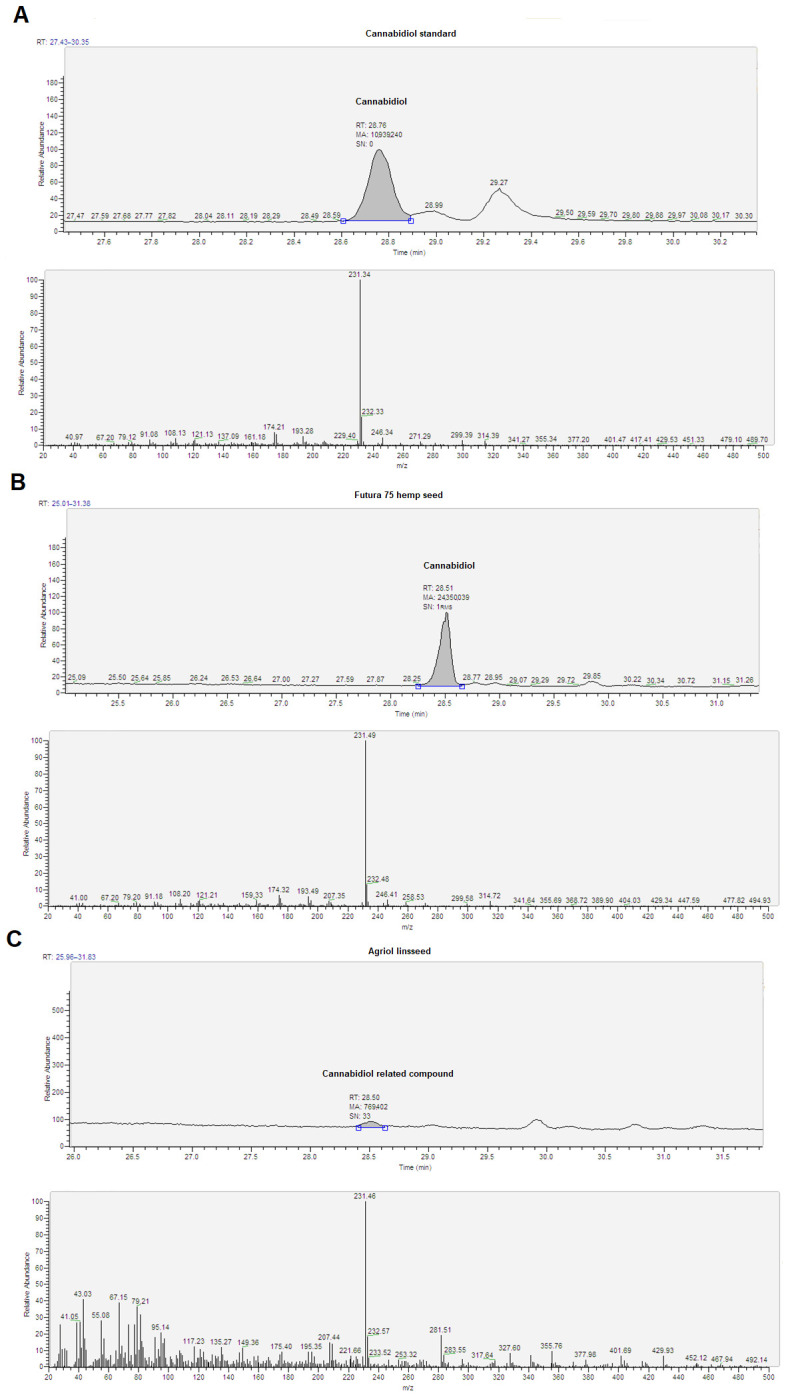
GC–MS chromatograms with mass spectra of the cannabidiol standard (**A**), a sample of the hemp seed variety Futura 75 (**B**) and the linseed variety Agriol (**C**) focused on the mass spectra of cannabidiol or cannabidiol related compound peak (retention time range 28.5–28.7 min).

In this study, 16 cannabinoid compounds were analyzed across all linseed varieties. Among these, only a compound exhibiting CBD-like chromatographic behavior (designated CBD*) was detected (Table 17). This putative CBD-like compound occurred at very low concentrations, with the highest level observed in the Bukoz variety (0.1137 mg/kg), while other varieties showed lower or non-detectable levels. A previous study by Styrczewska et al. [54] reported the presence of a cannabinoid-like compound in flax (*Linum usitatissimum* L.) with chromatographic and spectral characteristics similar to CBD. Using UPLC–MS, the authors observed comparable retention behavior and mass spectral fragments, suggesting the presence of a CBD-like metabolite, although its exact chemical identity was not confirmed. In the present study, HPLC-DAD analysis of the Agriol variety (Figure 2) revealed a signal with retention time and UV spectral characteristics similar to those of a CBD standard. Subsequent GC–MS analysis (Figure 3) showed comparable chromatographic behavior and mass spectral patterns when compared with both a CBD reference standard and a hemp seed sample (Futura 75). However, it must be emphasized that HPLC-DAD and GC–MS alone are not sufficient for definitive structural identification. Therefore, the detected compound can only be tentatively described as a putative CBD-like compound. Unambiguous confirmation of its chemical structure would require additional analytical techniques such as LC–MS/MS, high-resolution MS, or NMR spectroscopy. Taken together, these findings support the hypothesis that flax may contain secondary metabolites with chromatographic and spectral properties resembling cannabidiol. However, the compound cannot be conclusively identified as CBD, and its exact chemical nature and biological relevance remain to be elucidated.

### 3.6. Integrated Interpretation: A Functional Trade-Off Model Between Oilseed Crops

The results presented in Section 3.1, Section 3.2, Section 3.3, Section 3.4 and Section 3.5 demonstrate that hemp seed and linseed differ not only in individual compositional parameters but also in their overall functional profiles. Rather than representing isolated nutritional attributes, the observed differences reflect a broader trade-off between key compositional domains, including protein content, lipid yield, fatty acid composition, fiber content, and bioactive compound levels.

From a systems perspective, hemp seed can be characterized as a protein- and fiber-oriented matrix, whereas linseed represents a lipid- and n-3 fatty acid-oriented matrix. This distinction is supported by the higher crude protein (241 g/kg) and total dietary fiber content (237 g/kg) observed in hemp (Table 4), compared to the substantially higher fat content in linseed (342 g/kg; Table 5). These compositional differences may not be independent; rather, they likely reflect differences in metabolic allocation strategies under the studied conditions during seed development, where carbon and nitrogen resources are partitioned between storage proteins, structural polysaccharides, and lipids.

A key functional divergence is evident in fatty acid composition (Table 6, Table 7, Table 8, Table 9, Table 10 and Table 11). Linseed exhibits a markedly higher content of ALA and a more favorable n-6/n-3 ratio (1.18:1), which is associated with anti-inflammatory and cardioprotective effects. In contrast, hemp seed provides a more balanced fatty acid profile with additional bioactive components such as γ-linolenic acid, albeit with a less optimal n-6/n-3 ratio (3.56:1). This indicates that nutritional optimization cannot be reduced to a single parameter, but instead requires consideration of the overall lipid profile and its physiological implications.

Similarly, the higher levels of tocopherols and carotenoids in hemp seeds (Table 14 and Table 15) suggest a stronger intrinsic antioxidant system, which may contribute to enhanced oxidative stability despite a lower total lipid content. This highlights an important functional interaction between lipid quantity and antioxidant protection: seeds with lower oil content may compensate through higher concentrations of protective compounds, thereby maintaining lipid integrity.

The observed varietal variability across all measured parameters further supports the concept of a trade-off model. Significant differences (*p* < 0.05) in fatty acid composition, amino acid profiles, tocopherol levels, and cannabinoid content indicate that genotype-associated differences may enable targeted optimization of specific traits, potentially accompanied by trade-offs in other compositional parameters. For example, linseed varieties bred for low ALA (e.g., Agriol) exhibit altered fatty acid balance, while hemp varieties selected for specific cannabinoid profiles (e.g., Santhica types) show distinct secondary metabolite distributions.

Importantly, the cannabinoid analysis (Table 16 and Table 17) provides an additional dimension to this model. While hemp seeds contain only trace amounts of cannabinoids due to surface contamination rather than endogenous synthesis, the variability observed reflects whole-plant metabolic characteristics. In contrast, the detection of a CBD-like compound in linseed (CBD*), although at negligible concentrations, suggests the presence of structurally related secondary metabolites, further expanding the biochemical diversity of oilseed crops.

Taken together, these findings support a functional trade-off model, in which oilseed crops cannot simultaneously maximize all desirable nutritional and technological traits. Instead, hemp seed and linseed occupy distinct but complementary positions within a multidimensional compositional space. From an application perspective, this implies that: (i) linseed is better suited for applications requiring high oil yield and elevated n-3 fatty acid content, (ii) hemp seed is more suitable for protein enrichment, fiber intake, and antioxidant delivery.

Therefore, optimal utilization of these crops may not rely on selecting a single “superior” seed type, but rather on strategic combination or targeted varietal selection, depending on the intended nutritional or technological outcome. This integrative perspective addresses the complexity of seed composition and provides a framework for future research, breeding strategies, and food formulation approaches aimed at maximizing the functional potential of oilseed crops.

A limitation of the present study is that, due to the single-year experimental design, the relative contributions of genotype and environment cannot be fully disentangled; therefore, multi-year and multi-location evaluations would be required.

## 4. Conclusions

In this study, a comprehensive set of hemp and linseed varieties was evaluated using multiple complementary analytical approaches, including proximate composition, fatty acid profiling, amino acid composition, vitamin and carotenoid quantification, and cannabinoid analysis. In total, 12 hemp and 11 linseed varieties were analyzed in duplicates (independent biological replicates) and statistically evaluated (*p* < 0.05), allowing robust assessment of inter-varietal variability under standardized conditions.

The results indicated significant differences both between hemp and linseed and among individual varieties within each crop; however, while the study demonstrates considerable variation in composition under the tested conditions, it does not establish that these differences represent stable genetic traits independent of environmental influences. In terms of proximate composition, linseed exhibited higher total fat content, while hemp seeds showed slightly higher fiber content. Fat content ranged markedly among varieties, from approximately 200 g/kg to 377 g/kg, highlighting substantial varietal variability. The fatty acid profile of both crops was dominated by nutritionally important PUFAs, particularly linoleic acid (n-6) and ALA (n-3), with linseed demonstrating a more favorable n-6/n-3 ratio overall. Both crops also proved to be valuable sources of essential amino acids and bioactive compounds, including tocopherols and carotenoids (β-carotene, lutein, and zeaxanthin), which contribute to antioxidant potential and improve the nutritional stability of lipid-rich matrices. Considerable varietal differences were also observed in cannabinoid composition among hemp genotypes, including variability in CBD/CBDA and trace levels of Δ^9^-THC, although all analyzed hemp varieties complied with current EU regulatory limits for Δ9-THC equivalents, based on the applied calculation (Δ9-THC + 0.877 × Δ9-THCA), confirming that measured values remained below the 3.0 mg/kg threshold.

From an applied perspective, the results suggest that varietal selection may be used to influence the nutritional profile of hemp and linseed for feed purposes, particularly with respect to fatty acid composition and bioactive compound content. However, further studies evaluating digestibility, antinutritional factors, and in vivo animal performance are required to fully confirm the practical implications for feed formulation. The novelty of this study lies in the direct, side-by-side comparison of a relatively large number of commercially relevant hemp and linseed varieties in similar environmental conditions. This design enables a systematic assessment of varietal differences under uniform growing conditions and provides a unique, integrated dataset combining macronutrient composition, detailed lipid and amino acid profiles, micronutrients, and cannabinoid spectra within a single experimental framework. Overall, the findings indicate that both hemp and linseed are highly variable in their nutritional composition at the varietal level, and that this variability may be exploited in animal nutrition. While linseed remains superior in terms of total PUFA content and n-3/n-6 ratio, hemp seeds offer a broader spectrum of bioactive compounds, making both crops complementary rather than interchangeable functional feed ingredients. From a practical perspective, linseed may be considered the preferred option when a high content of oil and n-3 fatty acids is desired, whereas hemp seed appears more advantageous for applications emphasizing protein, fiber, and antioxidant compounds. To fully disentangle the relative contributions of genotype and environmental effects, multi-year and multi-location studies would be required.

## Figures and Tables

**Table 1:** Analyzed hemp seed and linseed varieties.

Seed	Variety	Origin	EU Registration	Vegetation Period (d)
Hemp seed	Białobrzeskie	Poland	Yes	135
Hemp seed	Carmagnola	Italy	Yes	160
Hemp seed	Dioica 88	France	Yes	150
Hemp seed	Earlina 8 FC	France	Yes	130
Hemp seed	Felina 32	France	Yes	115
Hemp seed	Ferimon 12	France	Yes	106
Hemp seed	Fibror 79	France	Yes	130
Hemp seed	Finola	Finland	Yes	95
Hemp seed	Futura 75	France	Yes	140
Hemp seed	Santhica 27	France	Yes	125
Hemp seed	Santhica 70	France	Yes	120
Hemp seed	USO 31	Ukraine	Yes	110
Linseed	Agram brown seeds MLA	Czech Republic	Yes	110
Linseed	Agriol yellow seeds LLA	Czech Republic	Yes	160
Linseed	Astella brown seeds HLA	Czech Republic	Yes	110
Linseed	Bethune brown seeds HLA	Canada	No	95
Linseed	Bukoz brown seeds HLA	Poland	Yes	112
Linseed	Floral brown seeds HLA	France	Yes	120
Linseed	Koral yellow seeds HLA	France	Yes	110
Linseed	Raciol yellow seeds MLA	Czech Republic	Yes	90
Linseed	Spring (genotype AGT 1/2020) brown seeds HLA	Czech Republic	No	110
Linseed	Szafir brown seeds HLA	Poland	Yes	120
Linseed	Winter (genotype AGT 2/2020) brown seeds HLA	Czech Republic	No	150

Abbreviations: HLA—High content of linolenic acid; MLA—Medium content of linolenic acid; LLA—Low content of linolenic acid.

**Table 2:** Climatic conditions at the Šumperk site from January (I.) to October (X.) 2021.

	I.	II.	III.	IV.	V.	VI.	VII.	VIII.	IX.	X.
Average temperature (°C)	−1.7	−1.2	0.5	5.9	11.5	18.7	19.6	16.4	13.7	7.5
Long-term average (%)	45.0	42.6	30.6	21.6	87.7	93.9	81.6	91.8	17.5	10.7
Total precipitation (mm)	19.5	96.2	35.1	4.1	57.8	136.5	83.8	164.5	94.1	73.7
Long-term total precipitation (mm)	55.4	39.0	44.1	36.3	68.7	82.6	77.5	74.4	51.8	45.7

**Table 3:** Agrochemical soil analysis—Spring 2021.

Location	N (mg/kg)	P (mg/kg)	Mg (mg/kg)	Ca (mg/kg)	pH	K (mg/kg)	K/Mg Ratio
Šumperk	1630	105.9	157.1	2498.7	6.5	150.7	1.0
Vikýřovice	1730	84.6	109.4	2348.0	5.9	173.1	1.6

**Table 4:** Chemical composition (g/kg) in different hemp seed varieties.

Variety	DM	Crude Protein	Fat	Total Dietary Fiber	Ash	NFE
Białobrzeskie	943 ± 1.1 ^bc^	224 ± 0.6 ^h^	200 ± 8.9 ^e^	243 ± 1.3 ^d^	44.4 ± 0.16 ^g^	232 ± 12.1 ^a^
Carmagnola	942 ± 3.8 ^c^	247 ± 1.1 ^bc^	267 ± 8.7 ^bc^	219 ± 0.4 ^gh^	59.3 ± 0.12 ^a^	149 ± 13.4 ^d^
Dioica 88	943 ± 0.1 ^bc^	248 ± 0.5 ^b^	298 ± 5.3 ^a^	229 ± 0.5 ^f^	42.1 ± 0.10 ^j^	126 ± 5.3 ^e^
Earlina 8 FC	946 ± 0.5 ^b^	250 ± 1.3 ^a^	236 ± 1.0 ^d^	249 ± 1.7 ^c^	43.3 ± 0.17 ^i^	168 ± 0.8 ^c^
Felina 32	949 ± 0.2 ^a^	247 ± 0.1 ^bc^	274 ± 5.2 ^bc^	221 ± 0.4 ^g^	41.7 ± 0.07 ^j^	167 ± 4.5 ^c^
Ferimon 12	944 ± 0.2 ^bc^	239 ± 1.0 ^e^	262 ± 0.9 ^c^	258 ± 2.3 ^b^	45.0 ± 0.22 ^f^	139 ± 0.1 ^de^
Fibror 79	948 ± 2.4 ^ab^	236 ± 0.5 ^f^	228 ± 0.9 ^d^	240 ± 0.6 ^e^	43.9 ± 0.04 ^h^	200 ± 3.4 ^b^
Finola	902 ± 0.9 ^d^	247 ± 0.7 ^bc^	278 ± 15.9 ^b^	219 ± 0.2 ^gh^	51.9 ± 0.04 ^c^	106 ± 15.9 ^f^
Futura 75	940 ± 0.5 ^c^	238 ± 0.9 ^e^	266 ± 0.9 ^bc^	217 ± 1.5 ^h^	56.3 ± 0.05 ^b^	163 ± 0.1 ^cd^
Santhica 27	943 ± 0.7 ^c^	246 ± 0.8 ^c^	261 ± 0.7 ^c^	230 ± 0.0 ^e^	45.9 ± 0.49 ^de^	160 ± 1.3 ^cd^
Santhica 70	941 ± 0.6 ^c^	243 ± 0.3 ^d^	255 ± 0.7 ^c^	273 ± 0.6 ^a^	45.5 ± 0.04 ^e^	124 ± 2.3 ^e^
USO 31	947 ± 0.3 ^ab^	229 ± 0.9 ^g^	238 ± 5.5 ^d^	244 ± 1.9 ^d^	46.0 ± 0.13 ^d^	191 ± 7.8 ^b^
Mean ± SD	941 ± 12.4	241 ± 8.1	255 ± 25.8	237 ± 17.2	47.1 ± 5.53	160 ± 35.0
SEM	2.5	1.7	5.3	3.5	1.13	7.1
*p*	<0.001	<0.001	<0.001	<0.001	<0.001	<0.001

Abbreviations: DM—Dry matter; NFE—Nitrogen-free extract; SD—Standard deviation; SEM—Standard error of the mean; ^a–j^ Values within a column with different superscripts differ significantly at *p* < 0.05.

**Table 5:** Chemical composition (g/kg) in different linseed varieties.

Variety	DM	Crude Protein	Fat	Total Dietary Fiber	Ash	NFE
Agram	947 ± 0.0	190 ± 0.0 ^g^	356 ± 0.2 ^b^	160 ± 1.7 ^cd^	36.4 ± 0.88 ^c^	205 ± 0.9 ^cd^
Agriol	944 ± 0.3	205 ± 0.7 ^c^	362 ± 20.6 ^ab^	148 ± 3.8 ^e^	36.3 ± 0.00 ^c^	193 ± 23.9 ^d^
Astella	954 ± 4.5	187 ± 0.5 ^h^	289 ± 0.8 ^e^	141 ± 7.8 ^e^	38.7 ± 0.11 ^ab^	298 ± 13.6 ^a^
Bethune	951 ± 2.0	199 ± 0.6 ^e^	352 ± 1.6 ^b^	174 ± 1.8 ^b^	38.9 ± 0.46 ^ab^	187 ± 5.4 ^de^
Bukoz	944 ± 0.8	191 ± 0.2 ^g^	344 ± 14.3 ^bc^	190 ± 0.6 ^a^	39.3 ± 0.01 ^a^	190 ± 1.4 ^de^
Floral	933 ± 24.7	199 ± 0.3 ^e^	372 ± 1.5 ^ab^	165 ± 4.6 ^c^	35.0 ± 0.05 ^cd^	162 ± 31.2 ^e^
Koral	963 ± 0.9	208 ± 0.1 ^b^	377 ± 3.7 ^a^	180 ± 3.3 ^b^	36.1 ± 0.00 ^cd^	161 ± 0.7 ^e^
Raciol	941 ± 0.1	212 ± 1.2 ^a^	324 ± 0.3 ^cd^	92 ± 0.8 ^g^	38.7 ± 0.07 ^b^	275 ± 1.6 ^ab^
Spring	958 ± 0.4	199 ± 0.2 ^e^	339 ± 1.3 ^bc^	157 ± 0.5 ^d^	37.7 ± 2.64 ^bc^	226 ± 2.3 ^c^
Szafir	959 ± 6.6	195 ± 2.1 ^f^	314 ± 1.7 ^d^	156 ± 1.8 ^d^	35.6 ± 0.03 ^cd^	259 ± 8.6 ^b^
Winter	958 ± 5.4	202 ± 0.6 ^d^	333 ± 0.7 ^c^	129 ± 0.4 ^f^	34.4 ± 0.03 ^d^	258 ± 6.2 ^b^
Mean ± SD	950 ± 10.6	199 ± 7.6	342 ± 26.1	154 ± 26.2	37.0 ± 1.79	219 ± 46.5
SEM	2.3	1.6	5.6	5.6	0.38	9.9
*p*	ns	<0.001	<0.001	<0.001	0.001	<0.001

Abbreviations: DM—Dry matter; NFE—Nitrogen-free extract; SD—Standard deviation; SEM—Standard error of the mean; ns—not significant; ^a–h^ Values within a column with different superscripts differ significantly at *p* < 0.05.

**Table 6:** Profile and composition of saturated fatty acids in different hemp seed varieties (mg/kg).

Variety	C 6:0	8:0	10:0	12:0	13:0	14:0	15:0	16:0	17:0	18:0	20:0	21:0	22:0	23:0	24:0
Białobrzeskie	1.08 ± 0.440 ^c^	62.9 ± 0.07 ^b^	3.83 ± 0.075 ^d^	5.47 ± 0.221 ^de^	1.81 ± 0.010	128 ± 1.1 ^e^	51.7 ± 1.40 ^f^	16,949 ± 9.2 ^k^	137 ± 1.8 ^d^	7634 ± 6.0 ^b^	2151 ± 37.5 ^g^	136 ± 1.9 ^de^	1.64 ± 0.216	0.375 ± 0.0842 ^bc^	9.2 ± 6.97 ^c^
Carmagnola	1.19 ± 0.027 ^c^	29.5 ± 1.48 ^e^	4.62 ± 0.314 ^c^	7.89 ± 0.251 ^c^	2.65 ± 0.592	171 ± 0.9 ^c^	72.6 ± 3.21 ^c^	22,541 ± 11.9 ^d^	171 ± 30.2 ^c^	4662 ± 160.8 ^fg^	2334 ± 53.5 ^f^	223 ± 8.7 ^b^	2.06 ± 1.372	0.560 ± 0.0330 ^bc^	87.7 ± 2.76 ^a^
Dioica 88	4.38 ± 1.183 ^a^	46.3 ± 1.32 ^d^	5.83 ± 0.012 ^b^	8.26 ± 0.080 ^c^	4.06 ± 0.409	167 ± 5.3 ^cd^	93.4 ± 1.31 ^a^	26,568 ± 7.3 ^a^	182 ± 1.0 ^bc^	6598 ± 149.0 ^d^	3016 ± 29.4 ^b^	221 ± 10.8 ^b^	2.28 ± 1.403	0.793 ± 0.2745 ^ab^	14.4 ± 7.54 ^c^
Earlina 8 FC	0.29 ± 0.062 ^c^	30.5 ± 1.90 ^e^	4.66 ± 0.077 ^c^	8.97 ± 0.158 ^b^	2.15 ± 0.341	153 ± 0.7 ^d^	71.1 ± 2.51 ^c^	20,014 ± 16.5 ^f^	193 ± 2.7 ^b^	6022 ± 18.8 ^e^	2560 ± 73.6 ^e^	137 ± 6.1 ^de^	2.80 ± 1.553	0.250 ± 0.0358 ^c^	43.8 ± 12.40 ^b^
Felina 32	0.76 ± 0.276 ^c^	43.4 ± 4.60 ^d^	3.64 ± 0.391 ^de^	3.94 ± 0.283 ^f^	2.94 ± 1.148	114 ± 0.2 ^fg^	57.9 ± 2.48 ^e^	18,943 ± 16.2 ^h^	157 ± 0.3 ^cd^	4830 ± 74.7 ^f^	2538 ± 83.3 ^e^	129 ± 4.4 ^e^	4.35 ± 4.413	0.216 ± 0.0379 ^c^	51.3 ± 15.78 ^b^
Ferimon 12	0.56 ± 0.163 ^c^	19.1 ± 0.04 ^f^	2.99 ± 0.194 ^e^	4.06 ± 0.077 ^f^	2.46 ± 0.356	122 ± 0.8 ^ef^	64.7 ± 2.15 ^d^	19,414 ± 11.4 ^g^	165 ± 5.7 ^c^	4377 ± 162.1 ^g^	2488 ± 62.9 ^e^	155 ± 7.3 ^d^	1.94 ± 0.898	0.338 ± 0.0112 ^c^	39.2 ± 20.10 ^bc^
Fibror 79	2.90 ± 0.170 ^b^	58.4 ± 0.20 ^b^	9.99 ± 0.148 ^cd^	6.03 ± 0.206 ^d^	2.33 ± 0.208	126 ± 0.4 ^ef^	69.0 ± 2.02 ^cd^	21,791 ± 27.9 ^e^	186 ± 3.4 ^bc^	8006 ± 187.4 ^a^	3122 ± 69.1 ^b^	187 ± 10.4 ^c^	2.97 ± 1.787	0.959 ± 0.0263 ^a^	50.5 ± 20.30 ^b^
Finola	3.84 ± 0.681 ^ab^	73.3 ± 2.60 ^a^	6.74 ± 0.000 ^a^	8.03 ± 0.027 ^c^	3.34 ± 1.587	181 ± 8.7 ^b^	85.6 ± 1.48 ^b^	24,371 ± 12.0 ^b^	245 ± 0.6 ^a^	6000 ± 56.3 ^e^	3372 ± 39.7 ^a^	330 ± 25.6 ^a^	4.27 ± 1.346	0.581 ± 0.0035 ^bc^	40.9 ± 1.07 ^bc^
Futura 75	1.45 ± 1.030 ^c^	47.4 ± 4.74 ^d^	6.61 ± 0.418 ^a^	9.96 ± 0.000 ^a^	1.65 ± 0.069	217 ± 1.7 ^a^	84.3 ± 0.51 ^b^	22,964 ± 0.4 ^c^	166 ± 0.6 ^c^	7284 ± 299.0 ^c^	2887 ± 19.9 ^c^	227 ± 2.8 ^b^	1.52 ± 0.244	0.640 ± 0.2503 ^b^	7.6 ± 0.85 ^c^
Santhica 27	2.64 ± 0.836 ^bc^	56.5 ± 1.61 ^c^	2.37 ± 0.179 ^e^	3.84 ± 0.358 ^f^	2.30 ± 0.586	110 ± 1.9 ^g^	56.7 ± 1.12 ^e^	16,517 ± 29.7 ^l^	134 ± 5.0 ^d^	4516 ± 203.6 ^fg^	1856 ± 55.4 ^h^	139 ± 10.8 ^de^	2.28 ± 1.141	0.400 ± 0.1506 ^bc^	31.2 ± 10.19 ^bc^
Santhica 70	1.02 ± 0.184 ^c^	70.7 ± 4.74 ^a^	2.28 ± 0.981 ^e^	5.01 ± 0.517 ^e^	2.50 ± 0.485	129 ± 0.4 ^e^	65.3 ± 0.46 ^d^	18,813 ± 0.0 ^i^	142 ± 0.7 ^d^	6373 ± 66.2 ^de^	2267 ± 80.8 ^fg^	155 ± 14.0 ^d^	2.44 ± 0.771	0.467 ± 0.0000 ^bc^	57.6 ± 19.36 ^b^
USO 31	2.07 ± 0.581 ^bc^	29.2 ± 0.26 ^e^	3.76 ± 0.143 ^de^	4.57 ± 0.493 ^ef^	2.99 ± 0.012	120 ± 0.2 ^f^	66.6 ± 1.18 ^d^	18,352 ± 3.5 ^j^	161 ± 5.2 ^cd^	6108 ± 185.5 ^e^	2733 ± 51.7 ^d^	153 ± 6.7 ^d^	0.73 ± 0.000	0.572 ± 0.1525 ^bc^	57.1 ± 16.53 ^b^
Mean ± SD	1.85 ± 1.366	47.3 ± 17.33	4.28 ± 1.479	6.34 ± 2.134	2.60 ± 0.806	145 ± 32.3	69.9 ± 12.32	20,602 ± 3066.3	170 ± 30.2	6034 ± 1237.0	2610 ± 436.2	183 ± 58.2	2.44 ± 1.589	0.513 ± 0.2328	40.9 ± 24.49
SEM	0.279	3.54	0.302	0.436	0.165	6.6	2.51	612.3	6.2	248.6	87.4	11.9	0.324	0.0475	5.00
*p*	<0.001	<0.001	<0.001	<0.001	ns	<0.001	<0.001	<0.001	<0.001	<0.001	<0.001	<0.001	ns	0.003	0.002

Abbreviations: SD—Standard deviation; SEM—Standard error of the mean; ns—not significant; ^a–l^ Values within a column with different superscripts differ significantly at *p* < 0.05.

**Table 7:** Profile and composition of saturated fatty acids in different linseed varieties (mg/kg).

Variety	C 6:0	8:0	10:0	12:0	13:0	14:0	15:0	16:0	17:0	18:0	20:0	21:0	22:0	23:0	24:0
Agram	3.88 ± 1.939 ^ab^	11.0 ± 1.14 ^bc^	5.63 ± 0.296 ^bc^	26.5 ± 2.43 ^bc^	3.37 ± 1.095	296 ± 28.2 ^ab^	109 ± 1.1 ^b^	32,466 ± 17.9 ^b^	268 ± 5.7 ^a^	10,505 ± 4262.1 ^a^	682 ± 2.0 ^c^	34.4 ± 1.48 ^ab^	11.40 ± 6.760	0.814 ± 0.2533	4.6 ± 0.52 ^c^
Agriol	5.02 ± 1.491 ^ab^	1.4 ± 0.51 ^d^	4.98 ± 0.199 ^c^	13.0 ± 0.31 ^d^	2.96 ± 1.371	152 ± 2.8 ^c^	51 ± 2.0 ^d^	17,250 ± 11.5 ^j^	182 ± 19.8 ^bc^	4166 ± 87.9 ^b^	382 ± 18.6 ^e^	17.1 ± 3.03 ^c^	1.24 ± 0.233	0.457 ± 0.0147	29.6 ± 6.28 ^b^
Astella	3.89 ± 0.581 ^ab^	12.7 ± 1.05 ^b^	4.89 ± 0.728 ^c^	23.6 ± 0.05 ^c^	2.78 ± 0.884	258 ± 22.7 ^ab^	98 ± 3.8 ^c^	28,978 ± 8.3 ^g^	247 ± 36.7 ^a^	8031 ± 48.9 ^ab^	715 ± 14.2 ^bc^	36.4 ± 7.55 ^ab^	4.40 ± 1.193	0.604 ± 0.0564	67.6 ± 7.81 ^a^
Bethune	1.63 ± 0.529 ^b^	9.5 ± 0.84 ^bc^	4.94 ± 0.629 ^c^	22.2 ± 1.40 ^c^	3.12 ± 0.559	259 ± 30.5 ^ab^	114 ± 7.8 ^b^	27,728 ± 38.0 ^h^	202 ± 18.9 ^b^	5606 ± 5.7 ^b^	689 ± 33.4 ^c^	34.0 ± 1.00 ^ab^	4.46 ± 2.390	0.462 ± 0.1662	7.1 ± 0.65 ^c^
Bukoz	2.25 ± 0.929 ^b^	11.3 ± 1.97 ^bc^	4.79 ± 0.054 ^c^	23.4 ± 2.33 ^c^	3.11 ± 0.531	249 ± 26.6 ^ab^	112 ± 3.7 ^b^	29,061 ± 24.7 ^f^	178 ± 8.2 ^bc^	5463 ± 49.6 ^b^	530 ± 30.3 ^d^	31.9 ± 4.36 ^ab^	3.19 ± 2.009	0.381 ± 0.0226	11.0 ± 10.35 ^c^
Floral	2.38 ± 0.207 ^b^	16.9 ± 0.75 ^a^	5.92 ± 0.395 ^b^	28.7 ± 0.09 ^b^	3.59 ± 0.239	299 ± 30.9 ^a^	131 ± 3.6 ^a^	34,040 ± 25.3 ^a^	217 ± 19.5 ^ab^	9026 ± 31.3 ^a^	863 ± 41.4 ^a^	33.8 ± 3.33 ^ab^	4.85 ± 1.535	0.503 ± 0.1634	6.7 ± 4.06 ^c^
Koral	5.24 ± 1.741 ^a^	10.8 ± 0.45 ^bc^	5.71 ± 0.103 ^bc^	22.8 ± 0.23 ^c^	3.47 ± 0.941	237 ± 33.4 ^ab^	132 ± 2.2 ^a^	30,754 ± 25.2 ^c^	270 ± 02.3 ^a^	10,170 ± 1300.3 ^a^	759 ± 19.4 ^bc^	29.3 ± 6.34 ^b^	5.21 ± 0.617	0.674 ± 0.4367	27.3 ± 7.92 ^b^
Raciol	4.05 ± 0.106 ^ab^	9.2 ± 0.09 ^c^	6.26 ± 0.311 ^b^	26.8 ± 1.90 ^bc^	3.37 ± 0.871	270 ± 29.4 ^ab^	109 ± 5.9 ^b^	29,269 ± 22.4 ^e^	265 ± 12.9 ^a^	8840 ± 760.9 ^a^	699 ± 06.6 ^c^	34.9 ± 1.42 ^ab^	4.18 ± 2.362	1.003 ± 0.3707	7.5 ± 3.34 ^c^
Spring	3.02 ± 0.757 ^b^	9.0 ± 2.30 ^c^	4.28 ± 0.338 ^c^	20.6 ± 0.74 ^c^	2.87 ± 0.241	232 ± 46.9 ^b^	92 ± 5.8 ^c^	26,003 ± 17.6 ^i^	192 ± 03.7 ^b^	7520 ± 193.1 ^ab^	865 ± 42.5 a	32.0 ± 1.93 ^ab^	8.82 ± 0.150	0.308 ± 0.0289	5.1 ± 4.25 ^c^
Szafir	1.54 ± 0.114 ^b^	11.7 ± 2.15 ^bc^	5.50 ± 0.275 ^bc^	23.8 ± 1.30 ^c^	3.51 ± 1.502	268 ± 42.9 ^ab^	123 ± 2.2 ^ab^	30,798 ± 20.3 ^c^	242 ± 12.9 ^a^	8292 ± 463.6 ^ab^	832 ± 38.6 ^a^	38.0 ± 1.10 ^a^	2.97 ± 0.709	0.820 ± 0.1386	5.4 ± 2.91 ^c^
Winter	2.85 ± 0.270 ^b^	15.8 ± 2.74 ^ab^	9.39 ± 0.248 ^a^	33.5 ± 3.02 ^a^	4.36 ± 1.743	278 ± 4.2 ^ab^	133 ± 8.1 ^a^	29,333 ± 4.5 ^d^	149 ± 1.8 ^c^	8267 ± 113.0 ^ab^	764 ± 23.5 ^b^	38.3 ± 3.47 ^a^	3.14 ± 0.741	1.094 ± 0.4511	28.2 ± 1.15 ^b^
Mean ± SD	3.25 ± 1.437	10.8 ± 4.08	5.66 ± 1.359	24.1 ± 5.15	3.32 ± 0.855	254 ± 44.8	110 ± 23.4	28,698 ± 4376.9	219 ± 42.3	7810 ± 218.1	707 ± 142.7	32.7 ± 6.31	4.90 ± 3.310	0.647 ± 0.3088	18.2 ± 19.15
SEM	0.306	0.87	0.290	1.10	0.182	9.6	5.0	911.3	9.0	46.5	30.4	1.35	0.706	0.0658	4.08
*p*	0.036	<0.001	<0.001	<0.001	ns	0.024	<0.001	<0.001	<0.001	0.013	<0.001	0.009	ns	ns	<0.001

Abbreviations: SD—Standard deviation; SEM—Standard error of the mean; ns—not significant; ^a–j^ Values within a column with different superscripts differ significantly at *p* < 0.05.

**Table 8:** Profile and composition of unsaturated fatty acids in different hemp seed varieties (mg/kg).

Variety	14:1-n5	16:1-n7	18:1-n9	18:1-n7	18:2-n6	18:3-n6	18:3-n3	18:2 (9,11)	18:2 (10,12)	20:1-n9	20:2-n6	20:3-n6	20:4-n6	20:3-n3	20:5-n3	22:1-n9	22:5-n3	24:1-n9	22:6-n3
Białobrzeskie	13.2 ± 0.38 ^c^	333 ± 4.9 ^f^	31,115 ± 9.4 ^h^	2049 ± 32.6 ^d^	133,525 ± 231.2 ^k^	7590 ± 9.67 ^h^	33,762 ± 16.6 ^l^	2365 ± 20.8 ^h^	18.3 ± 5.45	993 ± 5.9 ^f^	155 ± 7.9 ^e^	5.65 ± 3.111 ^bc^	0.94 ± 0.837	18.8 ± 2.81 ^d^	45.8 ± 6.06 ^de^	967 ± 6.7 ^f^	405 ± 67.1 ^b^	84.7 ± 14.25 ^de^	513 ± 46.2 ^d^
Carmagnola	10.7 ± 3.26 ^cd^	460 ± 10.0 ^b^	32,854 ± 2.8 ^f^	2418 ± 10.7 ^ab^	165,737 ± 111.1 ^f^	4347 ± 13.5 ^l^	54,599 ± 80.9 ^e^	2051 ± 20.5 ^i^	9.7 ± 2.53	1174 ± 27.5 ^cd^	193 ± 9.7 ^d^	4.73 ± 1.417 ^c^	0.76 ± 0.030	33.9 ± 4.01 ^bc^	117.5 ± 11.63 ^b^	1118 ± 6.6 ^d^	425 ± 21.1 ^b^	258.3 ± 14.79 ^a^	578 ± 13.3 ^cd^
Dioica 88	19.8 ± 0.27 ^ab^	472 ± 2.4 ^ab^	44,787 ± 17.9 ^a^	2468 ± 19.1 ^a^	225,650 ± 36.6 ^a^	4522 ± 5.0 ^k^	68,591 ± 48.7 ^a^	1750 ± 32.5 ^j^	27.2 ± 9.67	1650 ± 4.3 ^a^	255 ± 4.1 ^b^	5.80 ± 1.713 ^bc^	1.32 ± 0.006	40.9 ± 1.17 ^b^	76.2 ± 0.25 ^c^	1448 ± 13.4 ^a^	533 ± 13.6 ^a^	114.6 ± 1.38 ^c^	760 ± 2.3 ^a^
Earlina 8 FC	8.5 ± 0.54 ^d^	438 ± 14.9 ^c^	31,110 ± 4.2 ^h^	2356 ± 26.9 ^b^	171,149 ± 40.6 ^e^	10,245 ± 3.3 ^c^	56,840 ± 38.6 ^d^	3836 ± 9.2 ^b^	9.9 ± 4.72	1231 ± 37.2 ^c^	226 ± 12.6 ^c^	4.65 ± 0.161 ^c^	2.44 ± 1.613	29.7 ± 6.22 ^c^	35.8 ± 2.00 ^de^	1111 ± 7.8 ^d^	453 ± 14.0 ^ab^	68.5 ± 6.07 ^e^	740 ± 20.8 ^ab^
Felina 32	7.8 ± 0.48 ^d^	365 ± 7.2 ^e^	33,410 ± 15.1 ^e^	2209 ± 14.4 ^c^	157,737 ± 157.6 ^h^	8400 ± 35.2 ^e^	39,612 ± 69.8 ^k^	2609 ± 108.6 ^g^	14.3 ± 7.22	1155 ± 40.6 ^d^	189 ± 17.3 ^d^	4.64 ± 0.161 ^c^	1.86 ± 1.431	21.4 ± 2.27 ^d^	32.9 ± 6.70 ^e^	1124 ± 15.8 ^d^	453 ± 32.0 ^ab^	77.6 ± 11.14 ^de^	686 ± 29.9 ^b^
Ferimon 12	18.6 ± 2.15 ^b^	403 ± 18.5 ^d^	31,636 ± 47.3 ^g^	2292 ± 8.0 ^bc^	165,550 ± 39.0 ^f^	11,001 ± 7.0 ^b^	42,998 ± 457.5 ^i^	3242 ± 60.0 ^c^	13.7 ± 1.23	1164 ± 22.1 ^d^	204 ± 20.1 ^cd^	4.03 ± 1.655 ^c^	0.85 ± 0.416	22.4 ± 3.29 ^d^	48.7 ± 0.66 ^d^	1048 ± 8.0 ^e^	427 ± 22.8 ^b^	50.5 ± 2.75 ^ef^	753 ± 17.8 ^a^
Fibror 79	12.0 ± 0.58 ^cd^	484 ± 18.3 ^a^	39,979 ± 19.6 ^c^	2244 ± 22.8 ^c^	187,200 ± 153.4 d	5983 ± 19.7 ^j^	48,294 ± 57.0 ^f^	2146 ± 101.8 ^i^	9.9 ± 8.82	1223 ± 36.7 ^cd^	195 ± 12.5 ^d^	4.05 ± 1.787 ^c^	1.48 ± 1.103	22.5 ± 0.46 ^d^	74.7 ± 11.36 ^c^	1233 ± 8.3 ^b^	477 ± 67.5 ^ab^	45.1 ± 10.31 ^f^	691 ± 29.5 ^b^
Finola	23.3 ± 4.90 ^a^	443 ± 0.7 ^bc^	33,956 ± 79.7 ^d^	2492 ± 116.8 ^a^	203,837 ± 34.0 ^b^	16,852 ± 32.1 ^a^	67,428 ± 30.8 ^b^	5439 ± 37.1 ^a^	26.6 ± 2.14	1650 ± 30.2 ^a^	302 ± 13.8 ^a^	5.92 ± 5.357 ^a^	13.84 ± 11.726	48.5 ± 5.01 ^a^	203.0 ± 16.66 ^a^	1445 ± 1.3 ^a^	496 ± 26.9 ^ab^	165.5 ± 1.63 ^b^	606 ± 2.5 ^c^
Futura 75	18.9 ± 0.47 ^b^	472 ± 0.7 ^ab^	41,830 ± 14.1 ^b^	2250 ± 44.6 ^c^	196,529 ± 23.6 ^c^	6740 ± 8.6 ^i^	58,347 ± 11.7 ^c^	2407 ± 15.5 ^h^	17.8 ± 9.33	1389 ± 7.1 ^b^	233 ± 2.2 ^bc^	10.06 ± 0.863 ^b^	9.31 ± 1.483	28.1 ± 0.78 ^cd^	106.4 ± 1.15 ^b^	1221 ± 0.5 ^b^	454 ± 4.5 ^ab^	91.2 ± 6.05 ^d^	653 ± 3.3 ^bc^
Santhica 27	06.3 ± 0.15 ^d^	360 ± 8.8 ^e^	22,476 ± 14.4 ^j^	1991 ± 12.1 ^d^	136,888 ± 45.7 ^j^	7661 ± 26.1 ^g^	40,417 ± 60.1 ^j^	2774 ± 44.4 ^f^	11.3 ± 0.47	921 ± 39.1 ^g^	178 ± 13.5 ^de^	5.72 ± 2.158 ^bc^	0.96 ± 0.207	23.5 ± 2.74 ^cd^	51.1 ± 1.49 ^d^	815 ± 4.3 ^g^	347 ± 4.3 ^b^	77.0 ± 0.21 ^de^	536 ± 1.0 ^d^
Santhica 70	8.9 ± 0.17 ^d^	388 ± 3.4 ^de^	26,778 ± 45.2 ^i^	2208 ± 0.1 ^c^	156,832 ± 49.2 ^i^	8629 ± 24.8 ^d^	45,683 ± 55.8 ^h^	3036 ± 53.4 ^d^	15.5 ± 6.19	1072 ± 37.9 ^e^	198 ± 18.1 ^cd^	9.50 ± 0.052 ^b^	3.85 ± 2.598	26.7 ± 3.67 ^cd^	45.6 ± 1.81 ^de^	979 ± 10.6 ^f^	452 ± 81.3 ^ab^	78.9 ± 9.85 ^de^	606 ± 26.9 ^c^
USO 31	8.0 ± 0.29 ^d^	379 ± 4.2 ^e^	32,815 ± 28.6 ^f^	2020 ± 8.8 ^d^	160,414 ± 29.2 ^g^	8331 ± 8.0 ^f^	46,211 ± 36.3 ^g^	2900 ± 33.3 ^e^	14.7 ± 4.00	1222 ± 22.7 ^cd^	199 ± 13.6 ^cd^	2.88 ± 0.277 ^c^	0.52 ± 0.240	26.7 ± 1.79 ^cd^	46.3 ± 0.11 ^de^	1149 ± 1.5 ^c^	415 ± 23.6 ^b^	54.4 ± 18.58 ^ef^	729 ± 27.0 ^ab^
Mean ± SD	13.0 ± 5.74	416 ± 50.8	33,562 ± 6189.4	2250 ± 167.6	171,754 ± 27,113.0	8358 ± 3342.7	50,232 ± 11,015.0	2880 ± 986.0	15.7 ± 7.26	1237 ± 227.2	211 ± 39.2	7.30 ± 6.293	3.18 ± 4.803	28.6 ± 8.84	73.7 ± 48.08	1138 ± 184.6	445 ± 54.5	97.2 ± 59.26	654 ± 85.2
SEM	1.17	10.4	1236.5	34.6	5413.7	667.2	2199.6	197.3	1.48	46.4	8.0	1.285	0.980	1.81	9.81	37.5	11.1	12.10	17.4
*p*	<0.001	<0.001	<0.001	<0.001	<0.001	<0.001	<0.001	<0.001	ns	<0.001	<0.001	<0.001	ns	<0.001	<0.001	<0.001	0.048	<0.001	<0.001

Abbreviations: SD—Standard deviation; SEM—Standard error of the mean; ns—not significant; ^a–l^ Values within a column with different superscripts differ significantly at *p* < 0.05.

**Table 9:** Profile and composition of unsaturated fatty acids in different linseed varieties (mg/kg).

Variety	14:1-n5	16:1-n7	18:1-n9	18:1-n7	18:2-n6	18:3-n6	18:3-n3	18:2 (9,11)	18:2 (10,12)	20:1-n9	20:2-n6	20:3-n6	20:4-n6	20:3-n3	20:5-n3	22:1-n9	22:5-n3	24:1-n9	22:6-n3
Agram	4.65 ± 0.430 ^bc^	477 ± 6.1 ^b^	81,089 ± 12.2 ^f^	2962 ± 9.8 ^c^	133,735 ± 37.1 ^b^	44.2 ± 6.42 ^c^	216,382 ± 38.1 ^i^	153 ± 4.6 ^bc^	27.8 ± 3.72	586 ± 0.6 ^c^	374 ± 12.0 ^bc^	242 ± 0.1 ^cd^	14.36 ± 0.212 ^a^	172 ± 0.1 ^g^	36.1 ± 0.85 ^ab^	732 ± 3.2 ^cd^	455 ± 6.1 ^bc^	197 ± 2.2 ^d^	362 ± 4.5 ^c^
Agriol	3.88 ± 0.660 ^c^	290 ± 7.1 ^h^	41,556 ± 36.6 ^k^	2132 ± 23.3 ^g^	69,337 ± 596.9 ^f^	33.0 ± 3.21 ^cd^	6742 ± 11.5 ^j^	267 ± 2.1 ^a^	30.3 ± 2.19	314 ± 5.3 ^e^	174 ± 6.8 ^e^	9 ± 0.6 ^f^	0.68 ± 0.256 ^c^	3 ± 1.5 ^i^	33.5 ± 2.48 ^b^	326 ± 5.6 ^i^	266 ± 36.9 ^d^	51 ± 3.2 ^g^	710 ± 7.0 ^a^
Astella	6.53 ± 2.655 ^b^	408 ± 0.5 ^e^	65,039 ± 13.8 ^i^	2942 ± 68.4 ^cd^	61,581 ± 58.7 ^h^	48.9 ± 7.92 ^bc^	259,965 ± 160.1 ^e^	148 ± 2.5 ^bc^	44.3 ± 1.86	504 ± 29.0 ^d^	350 ± 9.1 ^c^	255 ± 8.3 ^c^	2.08 ± 1.325 ^bc^	249 ± 11.1 ^de^	14.1 ± 2.82 ^ef^	728 ± 14.4 ^d^	388 ± 59.8 ^c^	182 ± 2.3 ^e^	170 ± 1.7 ^e^
Bethune	3.23 ± 0.266 ^c^	382 ± 10.0 ^f^	92,183 ± 27.5 ^b^	2810 ± 10.8 ^e^	76,127 ± 24.4 ^e^	41.4 ± 11.72 ^cd^	269,595 ± 15.4 ^d^	148 ± 12.3 ^bc^	29.0 ± 1.74	784 ± 37.5 ^a^	394 ± 22.2 ^b^	260 ± 7.0 ^bc^	6.36 ± 1.764 ^b^	306 ± 1.6 ^b^	15.8 ± 3.00 ^ef^	684 ± 7.1 ^e^	463 ± 9.5 ^bc^	223 ± 4.0 ^b^	201 ± 4.6 ^d^
Bukoz	3.05 ± 0.016 ^c^	305 ± 7.8 ^g^	62,500 ± 39.6 ^j^	2785 ± 18.1 ^e^	84,330 ± 82.7 ^c^	37.5 ± 1.42 ^cd^	344,000 ± 85.3 ^a^	159 ± 9.3 ^bc^	22.9 ± 4.58	612 ± 24.7 ^bc^	359 ± 11.5 ^bc^	247 ± 3.2 ^cd^	4.41 ± 1.793 ^bc^	344 ± 1.6 ^a^	17.2 ± 1.47 ^e^	615 ± 0.1 ^g^	411 ± 11.7 ^c^	168 ± 0.0 ^f^	169 ± 0.5 ^e^
Floral	2.04 ± 0.540 ^c^	455 ± 9.6 ^cd^	84,351 ± 15.1 ^e^	2965 ± 1.6 ^c^	64,306 ± 52.2 ^g^	61.8 ± 3.44 ^b^	232,153 ± 43.8 ^g^	155 ± 23.8 ^bc^	37.2 ± 9.60	654 ± 33.8 ^b^	443 ± 38.4 ^a^	271 ± 1.3 ^b^	6.73 ± 3.044 ^b^	291 ± 0.7 ^c^	27.9 ± 1.40 ^c^	842 ± 13.7 ^a^	541 ± 11.7 ^a^	238 ± 1.5 ^a^	202 ± 0.5 ^d^
Koral	3.52 ± 0.408 ^c^	571 ± 0.1 ^a^	93,854 ± 51.2 ^a^	3496 ± 2.2 ^a^	57,537 ± 1014.0 ^i^	29.1 ± 0.42 ^d^	216,761 ± 47.2 ^h^	92 ± 27.2 ^d^	13.7 ± 11.67	603 ± 7.6 ^c^	337 ± 20.7 ^cd^	254 ± 9.6 ^c^	3.45 ± 1.453 ^bc^	258 ± 11.7 ^d^	17.6 ± 1.00 ^e^	517 ± 5.4 ^h^	456 ± 31.1 ^bc^	210 ± 6.5 ^c^	206 ± 8.4 ^d^
Raciol	3.89 ± 0.479 ^c^	437 ± 3.3 ^d^	71,478 ± 9.9 ^h^	2551 ± 37.4 ^f^	233,120 ± 21.4 ^a^	126.4 ± 8.82 ^a^	188,254 ± 57.0 ^k^	154 ± 03.7 ^bc^	21.7 ± 0.34	577 ± 4.0 ^c^	291 ± 12.0 ^d^	146 ± 2.9 ^e^	4.62 ± 3.466 ^bc^	155 ± 2.7 ^h^	39.4 ± 0.92 ^a^	633 ± 6.1 ^f^	464 ± 15.9 ^bc^	169 ± 2.3 ^f^	450 ± 6.9 ^b^
Spring	2.84 ± 0.534 ^c^	395 ± 2.2 ^f^	86,473 ± 59.6 ^d^	2895 ± 26.4 ^d^	76,436 ± 83.9 ^e^	38.0 ± 2.27 ^cd^	291,796 ± 10.7 ^c^	111 ± 35.0 ^cd^	14.7 ± 13.20	581 ± 25.7 ^c^	305 ± 12.2 ^d^	240 ± 3.7 ^d^	5.48 ± 5.837 ^bc^	209 ± 2.6 ^f^	10.3 ± 0.16 ^f^	814 ± 0.0 ^b^	501 ± 33.0 ^ab^	192 ± 1.4 ^d^	173 ± 1.3 ^e^
Szafir	5.00 ± 0.484 ^bc^	469 ± 4.8 ^bc^	80,301 ± 22.5 ^g^	3158 ± 22.9 ^b^	61,380 ± 20.6 ^h^	40.8 ± 9.66 ^cd^	301,285 ± 27.4 ^b^	138 ± 24.5 ^c^	30.5 ± 6.25	555 ± 26.6 ^c^	372 ± 19.5 ^bc^	242 ± 11.5 ^cd^	4.47 ± 0.024 ^bc^	255 ± 1.0 ^de^	13.5 ± 0.11 ^f^	747 ± 1.4 ^c^	481 ± 4.7 ^b^	238 ± 8.6 ^a^	165 ± 8.0 ^e^
Winter	10.58 ± 0.690 ^a^	459 ± 3.7 ^c^	87,807 ± 32.7 ^c^	2994 ± 40.2 ^c^	79,209 ± 27.3 ^d^	53.6 ± 1.88 ^bc^	244,113 ± 34.0 ^f^	180 ± 17.6 ^b^	33.7 ± 12.82	650 ± 13.3 ^bc^	366 ± 13.9 ^bc^	285 ± 7.2 ^a^	2.42 ± 1.115 ^bc^	246 ± 5.1 ^e^	24.1 ± 0.74 ^d^	835 ± 2.9 ^a^	488 ± 16.6 ^ab^	244 ± 0.9 ^a^	212 ± 1.0 ^d^
Mean ± SD	4.47 ± 2.398	422 ± 78.0	76,966 ± 15,629.7	2881 ± 342.1	90,645 ± 51,656.8	50.4 ± 26.60	251,910 ± 46,830.6	155 ± 45.2	27.8 ± 10.54	584 ± 112.8	342 ± 68.9	223 ± 77.6	5.01 ± 3.941	226 ± 90.2	22.7 ± 9.92	679 ± 150.1	447 ± 73.7	192 ± 52.9	275 ± 166.8
SEM	0.511	16.6	325.2	71.5	10,748.7	5.67	18,181.3	9.6	2.25	24.1	14.7	16.6	0.840	19.2	2.12	32.0	15.7	11.3	35.6
*p*	<0.001	<0.001	<0.001	<0.001	<0.001	<0.001	<0.001	<0.001	ns	<0.001	<0.001	<0.001	0.013	<0.001	<0.001	<0.001	<0.001	<0.001	<0.001

Abbreviations: SD—Standard deviation; SEM—Standard error of the mean; ns—not significant; ^a–k^ Values within a column with different superscripts differ significantly at *p* < 0.05.

**Table 10:** Summary of total, saturated, monounsaturated, polyunsaturated fatty acids, n-3 and n-6 fatty acids, and the n6/n3 ratio in different hemp seed varieties (g/kg).

Variety	Ʃ FA	Ʃ SFAs	Ʃ MUFAs	Ʃ PUFAs	Ʃ n-3	Ʃ n-6	n-6/n-3
Białobrzeskie	241.2 ± 0.50 ^j^	27.3 ± 0.07 ^g^	35.6 ± 0.06 ^i^	178.4 ± 0.41 ^l^	34.7 ± 0.13 ^l^	141.3 ± 0.25 ^l^	4.07 ± 0.008 ^a^
Carmagnola	296.7 ± 0.62 ^f^	30.3 ± 0.27 ^d^	38.3 ± 0.08 ^e^	228.1 ± 0.27 ^f^	55.8 ± 0.11 ^e^	170.3 ± 0.14 ^g^	3.05 ± 0.003 ^j^
Dioica 88	390.1 ± 0.83 ^a^	36.9 ± 0.10 ^a^	51.0 ± 0.05 ^a^	302.2 ± 0.14 ^a^	70.0 ± 0.06 ^a^	230.4 ± 0.03 ^a^	3.29 ± 0.002 ^g^
Earlina 8 FC	309.1 ± 0.33 ^e^	29.2 ± 0.01 ^e^	36.3 ± 0.10 ^h^	243.6 ± 0.13 ^e^	58.1 ± 0.08 ^d^	181.6 ± 0.06 ^e^	3.13 ± 0.003 ^i^
Felina 32	275.0 ± 0.61 ^i^	26.9 ± 0.06 ^g^	38.4 ± 0.11 ^e^	209.8 ± 0.46 ^j^	40.8 ± 0.13 ^k^	166.3 ± 0.21 ^i^	4.08 ± 0.008 ^a^
Ferimon 12	287.7 ± 0.80 ^g^	26.9 ± 0.27 ^g^	36.6 ± 0.09 ^g^	224.3 ± 0.29 ^g^	44.3 ± 0.41 ^i^	176.8 ± 0.07 ^f^	3.99 ± 0.039 ^b^
Fibror 79	323.9 ± 0.87 ^d^	33.6 ± 0.32 ^c^	45.2 ± 0.12 ^c^	245.1 ± 0.44 ^d^	49.6 ± 0.14 ^f^	193.4 ± 0.19 ^d^	3.90 ± 0.008 ^c^
Finola	370.2 ± 0.29 ^b^	34.7 ± 0.03 ^b^	40.2 ± 0.07 ^d^	295.3 ± 0.19 ^b^	68.8 ± 0.08 ^b^	221.0 ± 0.07 ^b^	3.21 ± 0.003 ^h^
Futura 75	346.7 ± 0.40 ^c^	33.9 ± 0.32 ^c^	47.3 ± 0.04 ^b^	265.5 ± 0.04 ^c^	59.6 ± 0.00 ^c^	203.5 ± 0.03 ^c^	3.42 ± 0.000 ^f^
Santhica 27	239.0 ± 0.59 ^k^	23.4 ± 0.32 ^h^	26.7 ± 0.08 ^k^	188.9 ± 0.19 ^k^	41.4 ± 0.07 ^j^	144.7 ± 0.08 ^k^	3.50 ± 0.003 ^e^
Santhica 70	275.1 ± 0.47 ^i^	28.1 ± 0.05 ^f^	31.5 ± 0.10 ^j^	215.5 ± 0.32 ^i^	46.8 ± 0.17 ^h^	165.7 ± 0.10 ^j^	3.54 ± 0.011 ^d^
USO 31	284.7 ± 0.51 ^h^	27.8 ± 0.27 ^f^	37.7 ± 0.07 ^f^	219.3 ± 0.18 ^h^	47.4 ± 0.09 ^g^	170.0 ± 0.05 ^h^	3.56 ± 0.006 ^d^
Mean ± SD	303.3 ± 46.22	29.9 ± 3.94	38.7 ± 6.51	234.7 ± 37.31	51.4 ± 10.88	180.3 ± 26.90	3.56 ± 0.360
SEM	9.44	0.80	1.33	7.62	2.22	5.49	0.073
*p*	<0.001	<0.001	<0.001	<0.001	<0.001	<0.001	<0.001

Abbreviations: SD—Standard deviation; SEM—Standard error of the mean; FA—fatty acids; SFAs—saturated fatty acids; MUFAs—monounsaturated fatty acids; PUFAs—polyunsaturated fatty acids; ^a–l^ Values within a column with different superscripts differ significantly at *p* < 0.05.

**Table 11:** Summary of total, saturated, monounsaturated, polyunsaturated fatty acids, n-3 and n-6 fatty acids, and the n6/n3 ratio in different linseed varieties (g/kg).

Variety	Ʃ FA	Ʃ SFAs	Ʃ MUFAs	Ʃ PUFAs	Ʃ n-3	Ʃ n-6	n-6/n-3
Agram	482.5 ± 4.07 ^e^	44.4 ± 4.20 ^a^	86.1 ± 0.03 ^f^	352.0 ± 0.10 ^e^	217.4 ± 0.05 ^i^	134.4 ± 0.06 ^b^	0.62 ± 0.000 ^c^
Agriol	144.5 ± 0.31 ^i^	22.3 ± 0.15 ^d^	44.7 ± 0.08 ^k^	77.6 ± 0.54 ^k^	7.8 ± 0.05 ^k^	69.6 ± 0.59 ^f^	8.97 ± 0.139 ^a^
Astella	431.5 ± 0.48 ^g^	38.5 ± 0.04 ^bc^	69.8 ± 0.13 ^i^	323.2 ± 0.31 ^h^	260.8 ± 0.23 ^e^	62.2 ± 0.08 ^h^	0.24 ± 0.000 ^de^
Bethune	479.3 ± 0.31 ^e^	34.7 ± 0.13 ^c^	97.1 ± 0.08 ^b^	347.6 ± 0.09 ^f^	270.6 ± 0.03 ^d^	76.8 ± 0.05 ^e^	0.28 ± 0.000 ^de^
Bukoz	532.8 ± 0.42 ^b^	35.7 ± 0.14 ^c^	67.0 ± 0.09 ^j^	430.1 ± 0.19 ^a^	344.9 ± 0.10 ^a^	85.0 ± 0.09 ^c^	0.25 ± 0.000 ^de^
Floral	432.7 ± 0.30 ^g^	44.7 ± 0.05 ^a^	89.5 ± 0.07 ^e^	298.5 ± 0.18 ^i^	233.2 ± 0.05 ^g^	65.1 ± 0.09 ^g^	0.28 ± 0.000 ^de^
Koral	417.7 ± 1.99 ^h^	42.4 ± 1.21 ^ab^	99.3 ± 0.07 ^a^	276.0 ± 0.85 ^j^	217.7 ± 0.10 ^h^	58.2 ± 0.99 ^i^	0.27 ± 0.005 ^de^
Raciol	538.6 ± 0.51 ^a^	39.6 ± 0.69 ^b^	75.9 ± 0.06 ^h^	423.2 ± 0.12 ^b^	189.4 ± 0.08 ^j^	233.7 ± 0.04 ^a^	1.23 ± 0.000 ^b^
Spring	496.2 ± 0.61 ^c^	35.0 ± 0.32 ^c^	91.4 ± 0.12 ^d^	369.8 ± 0.18 ^c^	292.7 ± 0.05 ^c^	77.0 ± 0.08 ^e^	0.26 ± 0.000 ^de^
Szafir	490.5 ± 0.78 ^d^	40.7 ± 0.58 ^b^	85.5 ± 0.09 ^g^	364.4 ± 0.11 ^d^	302.2 ± 0.04 ^b^	62.0 ± 0.04 ^h^	0.21 ± 0.000 ^e^
Winter	457.3 ± 0.10 ^f^	39.1 ± 0.06 ^b^	93.0 ± 0.03 ^c^	325.2 ± 0.13 ^g^	245.1 ± 0.06 ^f^	79.9 ± 0.05 ^d^	0.33 ± 0.000 ^d^
Mean ± SD	445.8 ± 104.77	37.9 ± 6.17	81.7 ± 15.80	326.15 ± 92.48	234.7 ± 85.24	91.3 ± 50.4	1.18 ± 2.540
SEM	22.34	1.32	3.37	19.72	18.17	10.75	0.541
*p*	<0.001	<0.001	<0.001	<0.001	<0.001	<0.001	<0.001

Abbreviations: SD—Standard deviation; SEM—Standard error of the mean; FA—fatty acids; SFAs—saturated fatty acids; MUFAs—monounsaturated fatty acids; PUFAs—polyunsaturated fatty acids; ^a–k^ Values within a column with different superscripts differ significantly at *p* < 0.05.

**Table 12:** Amino acid content (g/kg) in different hemp seed varieties.

Variety	Asp + Asn	Thr	Ser	Glu + Gln	Pro	Gly	Ala	Val	Ile	Leu	Tyr	Phe	His	Lys	Arg	Cys	Met
Białobrzeskie	23.6 ± 0.08 ^b^	7.38 ± 0.032 ^bc^	10.8 ± 0.17 ^bc^	39.4 ± 0.23 ^b^	9.18 ± 0.069 ^b^	9.85 ± 0.148 ^b^	9.88 ± 0.190 ^bc^	10.8 ± 0.14 ^bc^	8.92 ± 0.124 ^bc^	14.8 ± 0.24 ^bc^	6.71 ± 0.138 ^c^	10.30 ± 0.152 ^b^	6.34 ± 0.033 ^bc^	7.80 ± 0.123 ^bc^	26.1 ± 0.42 ^b^	3.84 ± 0.068 ^c^	5.25 ± 0.118 ^c^
Carmagnola	26.3 ± 1.21 ^a^	8.15 ± 0.329 ^a^	11.9 ± 0.51 ^a^	44.1 ± 2.08 ^a^	10.20 ± 0.459 ^a^	10.87 ± 0.451 ^a^	10.95 ± 0.529 ^a^	11.8 ± 0.55 ^a^	9.96 ± 0.438 ^a^	16.5 ± 0.73 ^a^	7.85 ± 0.421 ^a^	11.30 ± 0.471 ^a^	6.84 ± 0.306 ^a^	8.18 ± 0.286 ^b^	28.8 ± 1.28 ^a^	4.05 ± 0.071 ^bc^	5.74 ± 0.159 ^b^
Dioica 88	25.4 ± 0.51 ^a^	7.95 ± 0.307 ^ab^	11.5 ± 0.31 ^ab^	42.9 ± 1.13 ^ab^	9.37 ± 0.080 ^b^	10.68 ± 0.160 ^a^	10.53 ± 0.136 ^a^	11.3 ± 0.17 ^ab^	9.57 ± 0.173 ^ab^	16.0 ± 0.30 ^a^	7.25 ± 0.207 ^b^	10.90 ± 0.254 ^a^	6.77 ± 0.115 ^a^	8.83 ± 0.214 ^a^	28.0 ± 0.52 ^a^	4.46 ± 0.181 ^a^	5.77 ± 0.399 ^ab^
Earlina 8 FC	23.7 ± 0.30 ^b^	7.35 ± 0.004 ^bc^	10.9 ± 0.06 ^b^	39.1 ± 0.33 ^b^	8.59 ± 0.052 ^c^	9.79 ± 0.253 ^b^	9.87 ± 0.297 ^bc^	10.7 ± 0.24 ^bc^	8.97 ± 0.164 ^bc^	14.9 ± 0.28 ^bc^	6.62 ± 0.081 ^c^	10.23 ± 0.219 ^bc^	6.41 ± 0.116 ^b^	7.81 ± 0.115 ^bc^	26.3 ± 0.48 ^b^	4.30 ± 0.268 ^ab^	6.22 ± 0.328 ^a^
Felina 32	23.4 ± 0.02 ^b^	7.35 ± 0.051 ^bc^	10.6 ± 0.02 ^bc^	38.8 ± 0.16 ^bc^	8.95 ± 0.119 ^bc^	9.52 ± 0.044 ^bc^	9.57 ± 0.021 ^bc^	1.0.6 ± 0.01 ^bc^	8.73 ± 0.047 ^c^	14.5 ± 0.00 ^bc^	6.42 ± 0.031 ^cd^	10.17 ± 0.023 ^bc^	6.18 ± 0.037 ^bc^	7.58 ± 0.082 ^c^	25.3 ± 0.08 ^bc^	4.13 ± 0.051 ^bc^	5.87 ± 0.230 ^ab^
Ferimon 12	22.3 ± 0.35 ^c^	7.00 ± 0.032 ^cd^	10.1 ± 0.17 ^cd^	36.8 ± 0.81 ^c^	8.09 ± 0.005 ^d^	9.13 ± 0.213 ^c^	9.10 ± 0.197 ^c^	1.0.0 ± 0.14 ^cd^	8.33 ± 0.163 ^cd^	13.8 ± 0.27 ^cd^	6.06 ± 0.270 ^d^	9.56 ± 0.168 ^c^	6.09 ± 0.090 ^c^	7.42 ± 0.159 ^cd^	24.3 ± 0.76 ^c^	3.99 ± 0.019 ^bc^	5.83 ± 0.083 ^ab^
Fibror 79	20.7 ± 0.09 ^d^	6.55 ± 0.094 ^d^	9.4 ± 0.11 ^d^	34.5 ± 0.18 ^cd^	7.60 ± 0.054 ^e^	8.61 ± 0.025 ^d^	8.58 ± 0.015 ^d^	9.6 ± 0.02 ^d^	7.79 ± 0.052 ^d^	12.9 ± 0.09 ^d^	5.66 ± 0.050 ^d^	8.98 ± 0.052 ^d^	5.58 ± 0.031 ^d^	7.32 ± 0.035 ^cd^	22.8 ± 0.13 ^d^	4.11 ± 0.119 ^bc^	5.48 ± 0.063 ^bc^
Finola	23.6 ± 0.03 ^b^	7.55 ± 0.056 ^b^	11.0 ± 0.03 ^b^	39.6 ± 0.60 ^b^	8.54 ± 0.251 ^cd^	9.81 ± 0.158 ^b^	9.96 ± 0.089 ^b^	10.9 ± 0.35 ^b^	9.20 ± 0.068 ^b^	15.1 ± 0.01 ^b^	6.60 ± 0.109 ^c^	10.18 ± 0.170 ^bc^	6.16 ± 0.086 ^bc^	7.63 ± 0.283 ^c^	26.5 ± 0.22 ^b^	4.28 ± 0.218 ^ab^	6.01 ± 0.394 ^ab^
Futura 75	22.7 ± 0.60 ^bc^	7.06 ± 0.455 ^c^	10.2 ± 0.70 ^c^	37.4 ± 1.86 ^bc^	8.60 ± 0.364 ^c^	9.50 ± 0.349 ^bc^	9.43 ± 0.256 ^c^	10.2 ± 0.21 ^c^	8.54 ± 0.271 ^cd^	14.2 ± 0.47 ^c^	6.49 ± 0.172 ^cd^	9.73 ± 0.342 ^c^	6.12 ± 0.107 ^c^	7.97 ± 0.335 ^bc^	24.5 ± 0.91 ^c^	3.81 ± 0.185 ^c^	5.34 ± 0.112 ^bc^
Santhica 27	22.2 ± 0.01 ^c^	6.94 ± 0.017 ^cd^	10.0 ± 0.02 ^cd^	36.2 ± 0.01 ^cd^	8.09 ± 0.064 ^d^	9.10 ± 0.041 ^cd^	9.23 ± 0.014 ^c^	10.3 ± 0.13 ^c^	8.36 ± 0.021 ^cd^	13.8 ± 0.10 ^cd^	5.71 ± 0.256 ^d^	9.68 ± 0.020 ^c^	5.88 ± 0.104 ^cd^	7.39 ± 0.059 ^cd^	23.0 ± 0.31 ^cd^	3.83 ± 0.072 ^c^	5.12 ± 0.123 ^c^
Santhica 70	21.6 ± 0.67 ^cd^	6.58 ± 0.302 ^d^	9.4 ± 0.52 ^d^	34.4 ± 1.58 ^d^	7.68 ± 0.280 ^de^	8.80 ± 0.352 ^cd^	8.94 ± 0.377 ^cd^	10.0 ± 0.28 ^cd^	8.12 ± 0.331 ^d^	13.4 ± 0.52 ^d^	5.73 ± 0.326 ^d^	9.49 ± 0.331 ^c^	5.72 ± 0.194 ^d^	6.98 ± 0.189 ^d^	22.3 ± 1.19 ^d^	3.82 ± 0.027 ^c^	5.24 ± 0.105 ^c^
USO 31	21.6 ± 0.13 ^cd^	6.70 ± 0.140 ^cd^	9.8 ± 0.02 ^cd^	35.2 ± 0.40 ^cd^	7.97 ± 0.051 ^de^	8.86 ± 0.040 ^cd^	8.97 ± 0.105 ^cd^	9.8 ± 0.11 ^cd^	8.13 ± 0.048 ^d^	13.5 ± 0.03 ^d^	5.87 ± 0.151 ^d^	9.33 ± 0.008 ^cd^	5.89 ± 0.049 ^cd^	7.14 ± 0.128 ^d^	23.2 ± 0.13 ^cd^	4.14 ± 0.085 ^b^	5.91 ± 0.066 ^ab^
Mean ± SD	23.1 ± 1.62	7.21 ± 0.519	10.5 ± 0.80	38.2 ± 3.11	8.57 ± 0.759	9.54 ± 0.713	9.58 ± 0.699	10.5 ± 0.66	8.72 ± 0.641	14.5 ± 1.07	6.41 ± 0.671	9.99 ± 0.669	6.17 ± 0.385	7.67 ± 0.509	25.1 ± 2.12	4.06 ± 0.232	5.65 ± 0.377
SEM	0.33	0.106	0.16	0.64	0.155	0.145	0.143	0.13	0.131	0.22	0.137	0.137	0.079	0.104	0.43	0.047	0.077
*p*	<0.001	<0.001	<0.001	<0.001	<0.001	<0.001	<0.001	<0.001	<0.001	<0.001	<0.001	<0.001	<0.001	<0.001	<0.001	0.006	0.004

Abbreviations: SD—Standard deviation; SEM—Standard error of the mean; Asp—aspartic acid; Asn—asparagine; Thr—threonine; Ser—serine; Glu—glutamic acid; Gln—glutamine; Pro—proline; Gly—glycine; Ala—alanine; Val—valine; Ile—isoleucine; Leu—leucine; Tyr—tyrosine; Phe—phenylalanine; His—histidine; Lys—lysine; Arg—arginine; Cys—cysteine; Met—methionine; ^a–d^ Values within a column with different superscripts differ significantly at *p* < 0.05.

**Table 13:** Amino acid content (g/kg) in different linseed varieties.

Variety	Asp + Asn	Thr	Ser	Glu + Gln	Pro	Gly	Ala	Val	Ile	Leu	Tyr	Phe	His	Lys	Arg	Cys	Met
Agram	17.8 ± 0.53 ^bc^	6.47 ± 0.383 ^bc^	8.21 ± 0.528 ^bc^	33.9 ± 2.26 ^c^	6.82 ± 0.196 ^c^	11.2 ± 0.44 ^bc^	8.66 ± 0.256 ^bc^	9.30 ± 0.315 ^b^	7.78 ± 0.236 ^bc^	10.8 ± 0.39 ^cd^	4.53 ± 0.179 ^bc^	8.54 ± 0.244 ^cd^	4.56 ± 0.106 ^bc^	7.38 ± 0.350 ^b^	17.1 ± 0.47 ^bc^	3.37 ± 0.049 ^d^	4.11 ± 0.202
Agriol	18.0 ± 0.46 ^bc^	6.43 ± 0.203 ^bc^	8.48 ± 0.339 ^b^	36.3 ± 1.03 ^bc^	6.74 ± 0.119 ^cd^	11.1 ± 0.29 ^bc^	8.71 ± 0.261 ^bc^	9.19 ± 0.144 ^bc^	7.76 ± 0.215 ^bc^	11.0 ± 0.30 ^c^	4.56 ± 0.130 ^bc^	8.80 ± 0.252 ^c^	4.45 ± 0.106 ^c^	7.17 ± 0.217 ^bc^	17.3 ± 0.46 ^b^	3.88 ± 0.068 ^b^	4.41 ± 0.165
Astella	17.6 ± 0.23 ^bc^	6.50 ± 0.108 ^bc^	8.16 ± 0.086 ^bc^	35.5 ± 0.52 ^bc^	6.63 ± 0.224 ^cd^	10.7 ± 0.13 ^c^	8.20 ± 0.079 ^cd^	8.87 ± 0.160 ^c^	7.37 ± 0.098 ^cd^	10.4 ± 0.08 ^d^	4.11 ± 0.075 ^d^	8.19 ± 0.046 ^de^	4.26 ± 0.025 ^d^	7.01 ± 0.118 ^c^	16.6 ± 0.21 ^bc^	3.75 ± 0.033 ^bc^	4.28 ± 0.099
Bethune	18.3 ± 0.14 ^b^	6.70 ± 0.121 ^b^	8.51 ± 0.100 ^b^	37.2 ± 0.46 ^b^	6.72 ± 0.039 ^cd^	11.5 ± 0.11 ^b^	8.72 ± 0.093 ^bc^	9.44 ± 0.122 ^ab^	8.05 ± 0.063 ^b^	11.0 ± 0.06 ^c^	4.41 ± 0.038 ^c^	8.83 ± 0.047 ^c^	4.52 ± 0.021 ^bc^	7.49 ± 0.092 ^ab^	17.6 ± 0.59 ^b^	3.75 ± 0.142 ^bc^	4.26 ± 0.108
Bukoz	16.6 ± 0.20 ^c^	6.16 ± 0.010 ^c^	7.75 ± 0.022 ^c^	33.8 ± 0.35 ^c^	6.37 ± 0.101 ^d^	10.3 ± 0.18 ^c^	7.92 ± 0.177 ^d^	8.53 ± 0.205 ^c^	7.07 ± 0.131 ^d^	10.0 ± 0.11 ^d^	3.93 ± 0.082 ^d^	7.86 ± 0.084 ^e^	4.19 ± 0.072 ^d^	6.70 ± 0.120 ^cd^	16.1 ± 0.23 ^c^	3.56 ± 0.008 ^cd^	4.20 ± 0.114
Floral	19.7 ± 0.06 ^a^	7.31 ± 0.023 ^a^	9.03 ± 0.091 ^ab^	39.1 ± 0.10 ^ab^	7.16 ± 0.027 ^b^	11.7 ± 0.00 ^ab^	9.08 ± 0.021 ^ab^	9.77 ± 0.107 ^a^	8.36 ± 0.048 ^ab^	11.6 ± 0.12 ^b^	4.63 ± 0.033 ^b^	9.20 ± 0.003 ^b^	4.69 ± 0.073 ^b^	7.68 ± 0.009 ^ab^	18.6 ± 0.25 ^ab^	3.57 ± 0.017 ^c^	4.47 ± 0.071
Koral	17.1 ± 0.93 ^c^	6.30 ± 0.306 ^bc^	8.13 ± 0.225 ^bc^	36.2 ± 1.61 ^bc^	6.44 ± 0.154 ^d^	10.7 ± 0.11 ^c^	8.07 ± 0.052 ^d^	8.56 ± 0.168 ^c^	7.35 ± 0.115 ^d^	10.3 ± 0.17 ^d^	4.03 ± 0.084 ^d^	8.23 ± 0.180 ^d^	4.13 ± 0.063 ^d^	6.64 ± 0.070 ^d^	16.2 ± 0.84 ^c^	4.11 ± 0.088 ^a^	4.46 ± 0.099
Raciol	19.4 ± 0.05 ^a^	7.39 ± 0.008 ^a^	9.25 ± 0.065 ^a^	39.9 ± 0.35 ^a^	7.65 ± 0.094 ^a^	12.1 ± 0.07 ^a^	9.34 ± 0.090 ^a^	9.79 ± 0.010 ^a^	8.60 ± 0.054 ^a^	12.0 ± 0.11 ^a^	5.43 ± 0.090 ^a^	9.79 ± 0.060 ^a^	4.64 ± 0.089 ^bc^	7.74 ± 0.040 ^a^	18.6 ± 0.21 ^ab^	3.85 ± 0.057 ^bc^	4.32 ± 0.042
Spring	18.9 ± 0.46 ^ab^	6.74 ± 0.160 ^b^	8.38 ± 0.147 ^b^	36.7 ± 0.92 ^b^	6.50 ± 0.213 ^cd^	11.6 ± 0.25 ^ab^	9.02 ± 0.215 ^ab^	9.60 ± 0.140 ^ab^	8.01 ± 0.183 ^bc^	11.4 ± 0.22 ^bc^	4.24 ± 0.116 ^cd^	8.81 ± 0.194 ^c^	4.97 ± 0.099 ^a^	7.55 ± 0.130 ^ab^	16.9 ± 0.61 ^bc^	3.67 ± 0.235 ^c^	4.27 ± 0.243
Szafir	19.0 ± 0.24 ^ab^	7.04 ± 0.040 ^ab^	8.88 ± 0.065 ^ab^	39.2 ± 0.27 ^ab^	7.20 ± 0.082 ^b^	11.4 ± 0.13 ^b^	8.92 ± 0.046 ^b^	9.57 ± 0.221 ^ab^	8.30 ± 0.034 ^ab^	11.2 ± 0.08 ^bc^	4.57 ± 0.037 ^bc^	9.16 ± 0.047 ^bc^	4.53 ± 0.077 ^bc^	7.33 ± 0.045 ^bc^	18.8 ± 0.22 ^a^	3.74 ± 0.009 ^bc^	4.25 ± 0.139
Winter	17.9 ± 0.83 ^bc^	6.51 ± 0.403 ^bc^	8.33 ± 0.416 ^b^	37.2 ± 2.17 ^b^	6.35 ± 0.245 ^d^	11.1 ± 0.30 ^bc^	8.49 ± 0.211 ^c^	9.11 ± 0.293 ^bc^	7.70 ± 0.285 ^c^	10.7 ± 0.35 ^cd^	3.95 ± 0.040 ^d^	8.39 ± 0.296 ^d^	4.50 ± 0.137 ^c^	9.63 ± 0.180 ^cd^	16.8 ± 0.79 ^bc^	4.07 ± 0.029 ^ab^	4.10 ± 0.048
Mean ± SD	18.2 ± 1.01	6.69 ± 0.422	8.46 ± 0.464	36.8 ± 2.14	6.78 ± 0.414	11.2 ± 0.52	8.65 ± 0.447	9.25 ± 0.457	7.85 ± 0.472	11.0 ± 0.59	4.40 ± 0.426	8.71 ± 0.548	4.49 ± 0.241	7.24 ± 0.387	17.3 ± 1.02	3.76 ± 0.224	4.29 ± 0.157
SEM	0.22	0.090	0.099	0.46	0.088	0.11	0.095	0.097	0.101	0.13	0.091	0.117	0.051	0.082	0.22	0.048	0.033
*p*	0.001	0.002	0.003	0.004	<0.001	<0.001	<0.001	<0.001	<0.001	<0.001	<0.001	<0.001	<0.001	<0.001	0.001	<0.001	ns

Abbreviations: SD—Standard deviation; SEM—Standard error of the mean; ns—not significant; Asp—aspartic acid; Asn—asparagine; Thr—threonine; Ser—serine; Glu—glutamic acid; Gln—glutamine; Pro—proline; Gly—glycine; Ala—alanine; Val—valine; Ile—isoleucine; Leu—leucine; Tyr—tyrosine; Phe—phenylalanine; His—histidine; Lys—lysine; Arg—arginine; Cys—cysteine; Met—methionine; ^a–e^ Values within a column with different superscripts differ significantly at *p* < 0.05.

**Table 14:** Vitamin and carotenoid content (mg/kg) in different hemp seed varieties.

Variety	α-Tocopherol	γ-Tocopherol	δ-Tocopherol	Vitamin A	β-Carotene	Lutein	Zeaxanthin
Białobrzeskie	9.0 ± 0.06 ^i^	164 ± 1.1 ^f^	22.9 ± 0.23 ^f^	nd	0.279 ± 0.0021 ^e^	34.2 ± 0.28 ^d^	0.79 ± 0.006 ^f^
Carmagnola	14.3 ± 0.09 ^e^	179 ± 0.4 ^d^	26.1 ± 0.09 ^d^	nd	0.248 ± 0.0006 ^f^	24.3 ± 0.33 ^j^	0.49 ± 0.006 ^g^
Dioica 88	16.6 ± 0.38 ^c^	212 ± 1.0 ^a^	31.0 ± 0.10 ^b^	nd	0.403 ± 0.0033 ^c^	35.2 ± 0.23 ^c^	1.08 ± 0.032 ^d^
Earlina 8 FC	9.7 ± 0.11 ^h^	162 ± 0.5 ^g^	22.5 ± 0.17 ^g^	nd	0.338 ± 0.0024 ^d^	43.0 ± 0.12 ^a^	1.42 ± 0.029 ^a^
Felina 32	12.7 ± 0.22 ^g^	174 ± 0.5 ^e^	28.1 ± 0.26 ^c^	nd	0.278 ± 0.0004 ^e^	33.7 ± 0.12 ^e^	1.21 ± 0.006 ^b^
Ferimon 12	13.1 ± 0.14 ^f^	179 ± 0.9 ^d^	31.7 ± 0.19 ^a^	nd	0.220 ± 0.0024 ^h^	27.0 ± 0.14 ^h^	1.09 ± 0.005 ^d^
Fibror 79	17.9 ± 0.16 ^b^	113 ± 0.3 ^j^	15.9 ± 0.08 ^i^	nd	0.092 ± 0.0019 ^j^	13.5 ± 0.07 ^k^	0.40 ± 0.001 ^h^
Finola	19.5 ± 0.15 ^a^	179 ± 1.3 ^d^	26.3 ± 0.28 ^d^	nd	0.436 ± 0.0012 ^b^	35.2 ± 0.27 ^c^	1.19 ± 0.006 ^b^
Futura 75	14.8 ± 0.06 ^d^	183 ± 0.5 ^b^	31.2 ± 0.10 ^b^	nd	0.725 ± 0.0045 ^a^	37.4 ± 0.03 ^b^	1.13 ± 0.009 ^c^
Santhica 27	15.1 ± 0.12 ^d^	153 ± 0.1 ^i^	18.5 ± 0.09 ^h^	nd	0.248 ± 0.0000 ^f^	31.5 ± 0.22 ^f^	1.12 ± 0.004 ^cd^
Santhica 70	13.2 ± 0.15 ^f^	181 ± 0.8 ^c^	23.6 ± 0.13 ^e^	nd	0.232 ± 0.0023 ^g^	30.6 ± 0.37 ^g^	0.99 ± 0.005 ^e^
USO 31	10.1 ± 0.19 ^h^	158 ± 0.2 ^h^	18.6 ± 0.24 ^h^	nd	0.143 ± 0.0000 ^i^	25.0 ± 0.14 ^i^	1.09 ± 0.003 ^d^
Mean ± SD	13.8 ± 3.19	170 ± 22.9	24.7 ± 5.20	-	0.304 ± 0.1604	30.9 ± 7.46	1.00 ± 0.291
SEM	0.65	4.7	1.06	-	0.0327	1.52	0.059
*p*	<0.001	<0.001	<0.001	-	<0.001	<0.001	<0.001

Abbreviations: SD—Standard deviation; SEM—Standard error of the mean; nd—not detected; ^a–k^ Values within a column with different superscripts differ significantly at *p* < 0.05.

**Table 15:** Vitamin and carotenoid content (mg/kg) in different linseed varieties.

Variety	α-Tocopherol	γ-Tocopherol	δ-Tocopherol	Vitamin A	β-Carotene	Lutein	Zeaxanthin
Agram	1.25 ± 0.014 ^e^	121 ± 0.5 ^d^	5.34 ± 0.006 ^h^	nd	0.091 ± 0.0006 ^g^	2.14 ± 0.022 ^i^	0.400 ± 0.0065 ^g^
Agriol	1.65 ± 0.004 ^c^	145 ± 0.3 ^a^	7.09 ± 0.034 ^c^	nd	0.078 ± 0.0007 ^h^	1.15 ± 0.013 ^k^	0.250 ± 0.0017 ^i^
Astella	1.50 ± 0.007 ^d^	144 ± 0.2 ^a^	6.59 ± 0.012 ^e^	nd	0.139 ± 0.0002 ^c^	2.65 ± 0.027 ^g^	0.510 ± 0.0075 ^f^
Bethune	1.28 ± 0.007 ^e^	132 ± 0.3 ^b^	6.53 ± 0.038 ^e^	nd	0.121 ± 0.0008 ^d^	3.86 ± 0.031 ^b^	0.849 ± 0.0030 ^a^
Bukoz	2.09 ± 0.046 ^a^	124 ± 0.5 ^c^	6.75 ± 0.048 ^d^	nd	0.114 ± 0.0007 ^e^	3.75 ± 0.009 ^c^	0.737 ± 0.0032 ^b^
Floral	1.84 ± 0.032 ^b^	145 ± 0.1 ^a^	8.61 ± 0.059 ^a^	nd	0.195 ± 0.0021 ^a^	3.18 ± 0.012 ^e^	0.702 ± 0.0018 ^c^
Koral	0.94 ± 0.010 ^h^	115 ± 0.9 ^e^	6.04 ± 0.009 ^f^	nd	0.072 ± 0.0006 ^i^	1.52 ± 0.013 ^j^	0.301 ± 0.0023 ^h^
Raciol	1.08 ± 0.056 ^f^	132 ± 0.6 ^b^	7.82 ± 0.043 ^b^	nd	0.110 ± 0.0009 ^f^	2.88 ± 0.024 ^f^	0.584 ± 0.0053 ^e^
Spring	0.88 ± 0.004 ^i^	105 ± 0.3 ^f^	5.73 ± 0.039 ^g^	nd	0.109 ± 0.0006 ^f^	4.02 ± 0.081 ^a^	0.690 ± 0.0087 ^d^
Szafir	1.49 ± 0.015 ^d^	120 ± 0.8 ^d^	4.81 ± 0.013 ^j^	nd	0.193 ± 0.0013 ^a^	3.32 ± 0.059 ^d^	0.689 ± 0.0078 ^d^
Winter	1.01 ± 0.014 ^g^	106 ± 0.1 ^f^	5.18 ± 0.042 ^i^	nd	0.173 ± 0.0005 ^b^	2.55 ± 0.057 ^h^	0.581 ± 0.0022 ^e^
Mean ± SD	1.36 ± 0.379	126 ± 14.2	6.41 ± 1.124	-	0.128 ± 0.0423	2.82 ± 0.916	0.572 ± 0.1850
SEM	0.081	3.0	0.240	-	0.0090	0.195	0.0394
*p*	<0.001	<0.001	<0.001	-	<0.001	<0.001	<0.001

Abbreviations: SD—Standard deviation; SEM—Standard error of the mean; nd—not detected; ^a–k^ Values within a column with different superscripts differ significantly at *p* < 0.05.

**Table 16:** Cannabinoid content (mg/kg) in different hemp seed varieties.

Variety	CBDVA	CBDV	CBDA	CBGA	CBG	CBD	THCV	THCVA	CBN	Δ^9^-THC	Δ^8^-THC	CBNA	CBL	CBC	Δ^9^-THCA	CBCA	CBLA	Ʃ
Białobrzeskie	0.118 ± 0.0018 ^e^	nd	3.6 ± 0.18 ^h^	0.15 ± 0.001 ^g^	0.362 ± 0.0126 ^d^	1.63 ± 0.011 ^i^	nd	nd	nd	nd	nd	nd	nd	nd	nd	0.104 ± 0.0377 ^f^	0.685 ± 0.0208 ^ab^	6.6 ± 0.19 ^i^
Carmagnola	0.091 ± 0.0405 ^e^	nd	5.1 ± 0.08 ^g^	0.22 ± 0.056 ^g^	0.539 ± 0.0089 ^c^	6.33 ± 0.023 ^d^	nd	nd	0.224 ± 0.0023 ^c^	0.405 ± 0.0266 ^c^	nd	0.042 ± 0.0019 ^e^	nd	0.448 ± 0.0412 ^b^	nd	0.269 ± 0.0418 ^ef^	0.868 ± 0.1979 ^ab^	14.5 ± 0.35 ^g^
Dioica 88	0.293 ± 0.1098 ^de^	nd	14.0 ± 0.13 ^e^	0.52 ± 0.031 ^ef^	0.115 ± 0.0072 ^f^	5.02 ± 0.108 ^e^	nd	nd	nd	nd	nd	nd	nd	0.353 ± 0.0007 ^c^	nd	0.617 ± 0.0791 ^de^	0.676 ± 0.3052 ^ab^	21.6 ± 0.26 ^d^
Earlina 8 FC	0.469 ± 0.0348 ^d^	nd	9.1 ± 0.08 ^f^	0.44 ± 0.015 ^f^	0.022 ± 0.0044 ^g^	2.12 ± 0.051 ^h^	nd	nd	nd	nd	nd	0.070 ± 0.0034 ^d^	nd	0.171 ± 0.0604 ^d^	nd	0.437 ± 0.0419 ^e^	0.820 ± 0.2329 ^ab^	13.7 ± 0.22 ^h^
Felina 32	1.406 ± 0.0454 ^b^	nd	29.5 ± 0.20 ^c^	0.47 ± 0.000 ^ef^	0.217 ± 0.0782 ^e^	27.48 ± 0.193 ^a^	nd	nd	0.473 ± 0.0300 ^a^	0.575 ± 0.1442 ^b^	nd	0.223 ± 0.0093 ^a^	0.191 ± 0.0036 ^a^	0.532 ± 0.0192 ^b^	0.350 ± 0.0285 ^b^	1.172 ± 0.1372 ^c^	1.087 ± 0.2121 ^a^	63.7 ± 0.24 ^c^
Ferimon 12	0.371 ± 0.0032 ^de^	nd	14.9 ± 0.22 ^d^	0.55 ± 0.109 ^e^	0.095 ± 0.0004 f^g^	4.32 ± 0.077 ^g^	nd	nd	nd	nd	nd	0.137 ± 0.0023 ^b^	nd	0.147 ± 0.1064 ^d^	nd	0.567 ± 0.0860 ^de^	0.412 ± 0.1768 ^b^	21.5 ± 0.40 ^d^
Fibror 79	0.800 ± 0.0305 ^c^	nd	13.6 ± 0.04 ^e^	0.48 ± 0.003 ^ef^	0.106 ± 0.0257 ^f^	4.60 ± 0.048 ^f^	nd	nd	nd	nd	nd	0.087 ± 0.0254 ^c^	nd	0.478 ± 0.0436 ^b^	nd	0.636 ± 0.0903 ^d^	0.526 ± 0.3035 ^ab^	21.4 ± 0.32 ^d^
Finola	3.411 ± 0.4057 ^a^	nd	54.8 ± 1.09 ^a^	0.52 ± 0.066 ^ef^	0.568 ± 0.0009 ^c^	17.24 ± 0.082 ^b^	nd	nd	0.426 ± 0.0112 ^b^	1.946 ± 0.0000 ^a^	nd	nd	nd	0.283 ± 0.0027 ^cd^	nd	2.863 ± 0.0119 ^a^	nd	82.1 ± 0.56 ^a^
Futura 75	1.293 ± 0.0023 ^b^	nd	53.6 ± 0.55 ^b^	3.65 ± 0.021 ^c^	0.223 ± 0.0121 ^e^	10.98 ± 0.016 ^c^	nd	nd	0.051 ± 0.02673 ^d^	0.565 ± 0.0837 ^b^	nd	nd	0.059 ± 0.0111 ^b^	0.666 ± 0.0140 ^a^	0.603 ± 0.0749 ^a^	1.956 ± 0.2072 ^b^	0.639 ± 0.4262 ^ab^	74.2 ± 0.28 ^b^
Santhica 27	nd	nd	0.2 ± 0.00 ^k^	10.65 ± 0.080 ^b^	2.936 ± 0.0295 ^a^	0.32 ± 0.034 ^k^	nd	nd	nd	nd	nd	nd	nd	0.149 ± 0.0171 ^d^	nd	0.147 ± 0.0172 ^f^	0.937 ± 0.1266 ^a^	15.4 ± 0.04 ^f^
Santhica 70	nd	nd	1.1 ± 0.08 ^j^	11.81 ± 0.015 ^a^	2.453 ± 0.0281 ^b^	0.34 ± 0.032 ^k^	nd	nd	nd	nd	nd	nd	nd	0.216 ± 0.0161 ^d^	nd	0.111 ± 0.0233 ^f^	0.639 ± 0.0841 ^ab^	16.7 ± 0.01 ^e^
USO 31	0.022 ± 0.0008 ^e^	nd	2.4 ± 0.06 ^i^	0.16 ± 0.015 ^g^	0.035 ± 0.0192 ^g^	0.67 ± 0.001 ^j^	nd	nd	nd	nd	nd	0.013 ± 0.0001 ^f^	nd	nd	nd	0.090 ± 0.0100 ^f^	0.462 ± 0.0620 ^b^	3.9 ± 0.02 ^j^
Mean ± SD	0.689 ± 0.9680	-	16.8 ± 18.84	2.47 ± 4.114	0.639 ± 0.9607	6.75 ± 8.013	-	-	0.098 ± 0.1729	0.291 ± 0.5594	-	0.048 ± 0.0698	0.021 ± 0.0549	0.287 ± 0.2090	0.079 ± 0.1897	0.747 ± 0.8422	0.646 ± 0.3181	29.6 ± 26.62
SEM	0.1976	-	3.85	0.840	0.1961	1.636	-	-	0.0353	0.0112	-	0.0142	0.1112	0.0427	0.03871	0.1719	0.0649	5.43
*p*	<0.001	-	<0.001	<0.001	<0.001	<0.001	-	-	<0.001	<0.001	-	<0.001	<0.001	<0.001	<0.001	<0.001	0.023	<0.001

Abbreviations: SD—Standard deviation; SEM—Standard error of the mean; nd—not detected; the values are below the limit of detection (LOD) of the validated method described by Taubner and Czauderna [22], this publication provides the limits of detection (LOD) and limits of quantification (LOQ) for all cannabinoids analyzed in the present study; Δ^9^-THC—Δ^9^-tetrahydrocannabinol; Δ^9^-THCA—Δ^9^-tetrahydrocannabinolic acid; Δ^8^-THC—Δ^8^-tetrahydrocannabinol; CBD—cannabidiol; CBDA—cannabidiolic acid; CBN—cannabinol; CBNA—cannabinolic acid; CBG—cannabigerol; CBGA—cannabigerolic acid; CBC—cannabichromene; CBCA—cannabichromenic acid; THCV—Δ^9^-tetrahydrocannabivarin; THCVA—Δ^9^-tetrahydrocannabivarinic acid; CBDV—cannabidivarin; CBDVA—cannabidivarinic acid; CBL—cannabicyclol; ^a–k^ Values within a column with different superscripts differ significantly at *p* < 0.05.

**Table 17:** Content of putative CBD-like compound (mg/kg) (designed CBD*).

Variety	CBD*
Agram	nd
Agriol	0.111 ± 0.0031 ^b^
Astella	0.013 ± 0.000 ^g^
Bethune	0.009 ± 0.0025 ^h^
Bukoz	0.114 ± 0.0002 ^a^
Floral	0.051 ± 0.0020 ^e^
Koral	0.071 ± 0.0005 ^c^
Raciol	nd
Spring	0.012 ± 0.0001 ^g^
Szafir	0.055 ± 0.0015 ^d^
Winter	0.028 ± 0.0001 ^f^
Mean ± SD	0.042 ± 0.0406
SEM	0.0087
*p*	<0.001

Abbreviations: SD—Standard deviation; SEM—Standard error of the mean; nd—not detected; the values are below the limit of detection (LOD) of the validated method described by Taubner and Czauderna [22], this publication provides the limits of detection (LOD) and limits of quantification (LOQ) for all cannabinoids analyzed in the present study; CBD*—cannabidiol related compound; ^a–h^ Values within a column with different superscripts differ significantly at *p* < 0.05.

## Data Availability

The original contributions presented in this study are included in the article/Supplementary Materials. Further inquiries can be directed to the corresponding author.

## Supplementary Materials

The following supporting information can be downloaded at: https://www.mdpi.com/article/10.3390/foods15122145/s1, Table S1. Amino acid content (g/kg crude protein) in different hemp seed varieties. Table S2. Amino acid content (g/kg protein) in different linseed varieties.
