# Supplementary material for: Comparative Analysis of the Chemical Composition of Hemp and Linseed Varieties as Key Industrial Commodities
Source: Foods. 2026 Jun 14;15(12):2145. doi: 10.3390/foods15122145 (PMC13297758; doi:10.3390/foods15122145)
Supplement: Supplementary file 1 [file foods-15-02145-s001.zip › foods-4333540-supplementary.pdf]

Table S1. Amino acid content (g/kg crude protein) in different hemp seed varieties.

Variety	Asp + Asn	Thr	Ser	Glu + Gln	Pro	Gly	Ala	Val	Ile	Leu	Tyr	Phe	His	Lys	Arg	Cys	Met
Białobrzeskie	105.4	32.9	48.2	175.9	41.0	44.0	44.1	48.2	39.8	66.1	30.0	46.0	28.3	34.8	116.5	17.1	23.4
Carmagnola	106.5	33.0	48.2	178.5	41.3	44.0	44.3	47.8	40.3	66.8	31.8	45.7	27.7	33.1	116.6	16.4	23.2
Dioica 88	102.4	32.1	46.4	173.0	37.8	43.1	42.5	45.6	38.6	64.5	29.2	44.0	27.3	35.6	112.9	18.0	23.3
Earlina 8 FC	94.8	29.4	43.6	156.4	34.4	39.2	39.5	42.8	35.9	59.6	26.5	40.9	25.6	31.2	105.2	17.2	24.9
Felina 32	94.7	29.8	42.9	157.1	36.2	38.5	38.7	42.9	35.3	58.7	26.0	41.2	25.0	30.7	102.4	16.7	23.8
Ferimon 12	93.3	29.3	42.3	154.0	33.8	38.2	38.1	41.8	34.9	57.7	25.4	40.0	25.5	31.0	101.7	16.7	24.4
Fibror 79	87.7	27.8	39.8	146.2	32.2	36.5	36.4	40.7	33.0	54.7	24.0	38.1	23.6	31.0	96.6	17.4	23.2
Finola	95.5	30.6	44.5	160.3	34.6	39.7	40.3	44.1	37.2	61.1	26.7	41.2	24.9	30.9	107.3	17.3	24.3
Futura 75	95.4	29.7	42.9	157.1	36.1	39.9	39.6	42.9	35.9	59.7	27.3	40.9	25.7	33.5	102.9	16.0	22.4
Santhica 27	90.2	28.2	40.7	147.2	32.9	37.0	37.5	41.9	34.0	56.1	23.2	39.3	23.9	30.0	93.5	15.6	20.8
Santhica 70	88.9	27.1	38.7	141.6	31.6	36.2	36.8	41.2	33.4	55.1	23.6	39.1	23.5	28.7	91.8	15.7	21.6
USO 31	94.3	29.3	42.8	153.7	34.8	38.7	39.2	42.8	35.5	59.0	25.6	40.7	25.7	31.2	101.3	18.1	25.8
Mean	95.8	29.9	43.4	158.4	35.6	39.6	39.7	43.5	36.2	59.9	26.6	41.4	25.6	31.8	104.1	16.9	23.4

Abbreviations: Asp – aspartic acid; Asn – asparagine; Thr – threonine; Ser – serine; Glu – glutamic acid; Gln – glutamine; Pro – proline; Gly – glycine; Ala – alanine; Val – valine; Ile – isoleucine; Leu- leucine; Tyr – tyrosine; Phe – phenylalanine; His – histidine; Lys – lysine; Arg – arginine; Cys – cysteine; Met – methionine.

 https://doi.org/10.3390/foods15122145

Table S2. Amino acid content (g/kg protein) in different linseed varieties.

Variety	Asp + Asn	Thr	Ser	Glu + Gln	Pro	Gly	Ala	Val	Ile	Leu	Tyr	Phe	His	Lys	Arg	Cys	Met
Agram	93.7	34.1	43.2	178.4	35.9	58.9	45.6	48.9	40.9	56.8	23.8	44.9	24.0	38.8	90.0	17,7	21.6
Agriol	87.8	31.4	41.4	177.1	32.9	54.1	42.5	44.8	37.9	53.7	22.2	42.9	21.7	35.0	84.4	18,9	21.5
Astella	94.1	34.8	43.6	189.8	35.5	57.2	43.9	47.4	39.4	55.6	22.0	43.8	22.8	37.5	88.8	20,1	22.9
Bethune	92.0	33.7	42.8	186.9	33.8	57.8	43.8	47.4	40.5	55.3	22.2	44.4	22.7	37.6	88.4	18,8	21.4
Bukoz	86.9	32.3	40.6	177.0	33.4	53.9	41.5	44.7	37.0	52.4	20.6	41.2	21.9	35.1	84.3	18,6	22.0
Floral	99.0	36.7	45.4	196.5	36.0	58.8	45.6	49.1	42.0	58.3	23.3	46.2	23.6	38.6	93.5	17,9	22.5
Koral	82.2	30.3	39.1	174.0	31.0	51.4	38.8	41.2	35.3	49.5	19.4	39.6	19.9	31.9	77.9	19,8	21.4
Raciol	91.5	34.9	43.6	188.2	36.1	57.1	44.1	46.2	40.6	56.6	25.6	46.2	21.9	36.5	87.7	18,2	20.4
Spring	95.0	33.9	42.1	184.4	32.7	58.3	45.3	48.2	40.3	57.3	21.3	44.3	25.0	37.9	84.9	18,4	21.5
Szafir	97.4	36.1	45.5	201.0	36.9	58.5	45.7	49.1	42.6	57.4	23.4	47.0	23.2	37.6	96.4	19,2	21.8
Winter	88.6	32.2	41.2	184.2	31.4	55.0	42.0	45.1	38.1	53.0	19.6	41.5	22.3	47.7	83.2	20,1	20.3
Mean	91.7	33.7	42.6	185.2	34.1	56.5	43.5	46.6	39.5	55.1	22.1	43.8	22.6	37.7	87.2	18,9	21.6

Abbreviations: Asp – aspartic acid; Asn – asparagine; Thr – threonine; Ser – serine; Glu – glutamic acid; Gln – glutamine; Pro – proline; Gly – glycine; Ala – alanine; Val – valine; Ile – isoleucine; Leu- leucine; Tyr – tyrosine; Phe – phenylalanine; His – histidine; Lys – lysine; Arg – arginine; Cys – cysteine; Met – methionine.
